# Impact of ovalbumin allergy on oral and gut microbiome dynamics in 6-week-old BALB/c mice

**DOI:** 10.3389/fmicb.2024.1439452

**Published:** 2024-09-03

**Authors:** Chuanyue Qiao, Shuang Bian, Hao Huang, Han Xiao, Lei Ma, Rui Han

**Affiliations:** ^1^Department of Prosthodontics, The Affiliated Hospital of Qingdao University, Qingdao, China; ^2^School of Stomatology, Qingdao University, Qingdao, China; ^3^Department of Stomatology, Traditional Chinese Medical Hospital of Huangdao District, Qingdao, China; ^4^Department of Stomatology, Qingdao Eighth People's Hospital, Qingdao, China

**Keywords:** food allergy, ovalbumin, gut microbiota, oral microbiota, 16S rRNA gene sequencing, NOD-like receptor signaling pathway

## Abstract

**Background:**

The gut microbiota is known to have a significant impact on the development of food allergy, and several recent studies have suggested that both oral microbiota, which first come into contact with allergenic foods, may have a profound influence on the development of food allergy.

**Methods:**

In this study, we have established an ovalbumin-sensitive mice model by utilizing ovalbumin as a sensitizing agent. Subsequently, we performed a comprehensive analysis of the gut and oral microbiota in ovalbumin-sensitive mice and the control mice using full-length 16S rRNA sequencing analysis.

**Results:**

Interestingly, both the gut and oral microbiota of ovalbumin-sensitized mice exhibited significant dysbiosis. The relative abundance of *s__Lactobacillus_intestinalis* in the gut microbiota of ovalbumin-sensitive mice exhibited a significant decrease, whereas the abundance of *s__Agrobacterium_radiobacter* and *s__Acinetobacter_sp*__CIP_56_2 displayed a significant increase. Furthermore, the relative abundance of *s__unclassified_g__Staphylococcus*, *s__Streptococcus_hyointestinalis*, and *s__unclassified_g__Dechloromonas* in the oral microbiota of ovalbumin-sensitive mice revealed a significant decrease. In contrast, the abundance of 63 other species, including *s__Proteiniclasticum_ruminis*, *s__Guggenheimella_bovis*, and *s__Romboutsia_timonensis*, demonstrated a significant increase. The random forest classifier achieved the best accuracy in predicting the outcome of food allergy using three gut and three oral biomarkers, with accuracies of 94.12 and 100%, respectively. Based on the predictions of the PICRUSt2 analysis, the only consistent finding observed across multiple samples from both the groups of mice was a significant up-regulation of the nucleotide-binding oligomerization domain (NOD)-like receptor signaling pathway in the ovalbumin-sensitized mice.

**Conclusion:**

Our study demonstrates that ovalbumin-sensitized mice experience substantial alterations in both gut and oral microbial composition and structure, and specific strains identified in this study may serve as potential biomarkers for food allergy screening. Moreover, our findings highlight that the oral environment, under the same experimental conditions, exhibited greater precision in detecting a larger number of species. Additionally, it is worth noting that the NOD-like receptor signaling pathway plays a vital role in the pathogenesis of OVA (ovalbumin)-induced allergy. These findings will generate novel concepts and strategies in the realm of food allergy prevention and treatment.

## 1 Introduction

Food allergy (FA) is a detrimental health condition that results from a reproducible, Immunoglobulin E (IgE)-mediated, or non-IgE-mediated immune response upon exposure to certain food antigens (Ho and Bunyavanich, 2018). The prevalence of allergic diseases has been consistently increasing on a global scale, impacting up to 8% of children and 3% of adults in developed nations, with food allergy representing a major problem due to its severe clinical manifestations and its persistence into adulthood beyond childhood (Goldberg et al., 2020). Eggs are considered as one of the top eight allergenic foods, and egg allergy is the second most prevalent FA among newborns and toddlers, following cow’s milk allergy (Nwaru and Sheikh, 2012). The primary source of allergens in eggs is ovalbumin (OVA) found in the egg white, and allergic reactions triggered by OVA usually last for a long time (Savage et al., 2007). Studies have demonstrated that ingested allergens are absorbed by the gastrointestinal tract through paracellular diffusion and are subsequently internalized by antigen-presenting cells (APC), thereby triggering the activation of the Th2 response (Georas et al., 2005). This can lead to an array of allergic symptoms, such as itching, swelling of the tongue, hives, difficulty breathing, itchiness, abdominal pain, vomiting, diarrhea, urticaria, hypotension, and potentially life-threatening anaphylactic shock (Ben-Shoshan and Clarke, 2011).

The increasing prevalence of FA highlights the significant role of environmental factors in pathogenesis of the illness, with microbial exposure emerging as a pivotal environmental risk factor (Ho and Bunyavanich, 2018; Bunyavanich and Berin, 2019). In the early stages of life, it is widely acknowledged that intricate interactions among the gut microbiota, dietary antigens, intestinal inflammation, and the innate immune system exert a significant influence on whether an individual develops a robust tolerance or manifests FA (Fung et al., 2017). There is increasing evidence indicating that the gut microbiome plays a pivotal role in the onset of FA, both those mediated by IgE and not mediated by IgE (Berni Canani et al., 2018; Dong et al., 2018). Dysbiosis, or an imbalance in gut microbiota composition and function, can disrupt the integrity of the intestinal epithelial barrier, thereby facilitating the entry of antigens into the bloodstream as well as abnormal stimulation of the immune system, leading to disturbances in gut homeostasis and the promotion of disease (Bunyavanich and Berin, 2019; Wilkins et al., 2019; De Filippis et al., 2021). In addition, the early-life gut microbiome composition may hold predictive value in resolving FA. For instance, the researchers observed a positive correlation between elevated levels of *Clostridia* difficile in the intestines of children with cow’s milk allergy (CMA) and their increased likelihood to develop immune tolerance by the age of 8 (Bunyavanich et al., 2016). A recent study revealed that *Clostridia* can enhance the accumulation of regulatory T cells in the colonic lamina propria of mice, while concurrently reducing the production of specific Immunoglobulin E (sIgE) in the sera of OVA-allergic mice (Atarashi et al., 2011). *Clostridia* have been reported to modulate the composition of the commensal gastrointestinal microbiota, resulting in reduction of Th2-type cytokines production and attenuated allergic reaction by stimulating the generation of IL-22 (interleukin 22) in mice serum (Stefka et al., 2014). Moreover, oral administration of *L. plantarum* JC7 has shown the ability to prevent food allergy by regulating imbalances in Th1/Th2 and Treg/Th17, as well as modulating the dysregulated intestinal microbiota (Duan et al., 2023). Similarly, *Lactobacillus plantarum* IM76 and *Bifidobacterium longum* IM55 have demonstrated the ability to relieve OVA-induced allergic rhinitis in mice by promoting the restoration of intestinal microbial balance and regulation of Th2/Treg imbalance (Kim et al., 2019). Thus, manipulating the gut microbiome holds significant promise as an innovative approach for preventive and therapeutic strategies against allergies. It is essential to identify microbial markers linked to allergic conditions and comprehend the functional capabilities of the disturbed microbiome to develop innovative strategies in this field.

Although numerous studies have explored the role of the gut microbiome in food allergy, the oral microbiome, an equally crucial player, remains underexplored. The oral microbial community continues to develop with the emergence of primary teeth in early infancy and establishment of permanent dentition in children (Consortium HMP, 2012). This evolution leads to a diverse and intricate microbiome that is closely linked to the gut microbiota in both early stages of life and disease (Escapa et al., 2018; Xiao et al., 2020). Recent studies have indicated that variations in the salivary microbiome of young children are associated with iron deficiency anemia and caries. Specifically, oral flora of the iron deficiency anemia group displayed a wide variety of microorganisms in their mouth, including an increased presence of bacterial genera such as *Bacillus*, *Moraxella*, and *Rhodococcus*, leading to a higher prevalence of caries (Han et al., 2021). In addition, the oral mucosa represents the beginning of a continuous gastrointestinal mucosal system enriched with immune cells and microbiota; thus, the oral microbiota and metabolites produced by them could influence FA (Zhang et al., 2022). For instance, researchers have reported a significant decrease in the number of hydrogen peroxide-producing *Lactobacillus* sp. and a reduction in the diversity of oral commensal bacteria in the oral cavities of allergic mice, as observed in an OVA allergy murine model (Matsui et al., 2019). The oral microbiome of individuals with peanut allergies showed a decrease in certain species of bacteria in the orders *Lactobacillales*, *Bacteroidales* (*Prevotella spp*), and *Bacillales*, and increased *Neisseriales* spp., but was accompanied by significant reductions in oral short-chain fatty acid (SCFA) levels, including acetate, butyrate, and propionate (Ho et al., 2021). More interestingly, a recent study reported that microbiota and metabolites in the oral and gut environments were associated with reaction thresholds in challenge-proven FA. The researchers discovered distinct patterns of saliva microbiota that distinguished children with high-threshold (HT) peanut allergy from those with low-threshold (LT) peanut allergy (Zhang et al., 2022). Furthermore, they observed that LT children exhibited higher α-diversity in their saliva microbiome, whereas the HT group had a higher concentration of oral *Veillonella nakazawae* bacteria compared to the LT group (Zhang et al., 2022). However, until now, the research on the relationship between the oral microbiome and FA has been limited to these studies and there is a lack of thorough research on the oral host-commensal milieu in relation to FA.

Meanwhile, studies investigating the potential relationship between FA and microbiota primarily employ second-generation 16SrRNA sequencing methods. This approach can only target 1–2 regions from the nine variable regions of bacteria, thus limiting the ability to differentiate species at the species level and focusing mainly on microbial composition at the phylum-genera level (Johnson et al., 2019). In contrast, third-generation full-length 16SrRNA sequencing covers regions V1-V9, enabling a thorough analysis of the microbial composition at both the phylum-genera and species levels; amplicon sequencing based on three-generation sequencing technologies, such as PacBio, enables high-precision sequencing of the full-length sequences of 16S rRNA genes (Fuks et al., 2018). Second, unlike second-generation amplicon sequencing, which can bias the annotation accuracy of samples from different sources and species compositions (Johnson et al., 2019), full-length 16S amplicon sequencing has widespread applicability to a broader range of samples and species as it sequences the full-length sequence of the 16S rRNA gene (Fuks et al., 2018), avoiding the preference for different sample sources and species resulting from the sequencing of different regions as done in conventional amplicon sequencing (Pootakham et al., 2017). Therefore, this study employed a state-of-the-art third-generation full-length 16S rRNA sequencing technique to extensively investigate and analyze the gut and oral microbiomes of ovalbumin-allergic mice. This sequencing offers a higher accuracy rate for species-level identification, thereby increasing the confidence of the results. In summary, unraveling subtle changes in both the oral and gut microbiomes could pave the way for novel diagnostic and therapeutic strategies in managing food allergy.

## 2 Materials and methods

### 2.1 Animals and diets

Female BALB/c mice (SPF, Six-week-old, weight of 16–20 g) were purchased from Jinan Pengyue Experimental Animal Breeding Co., Ltd., (Jinan, China) and kept in the Experimental Animal Center of Affiliated Hospital of Qingdao University. The animals were housed in a controlled environment with a temperature maintained at 21 ± 2°C and humidity levels between 50 and 55%. The light/dark cycle was reversed, with lights on from 7.00 am until 7.00 pm, following a strict 12:12 h schedule. A total of 20 mice were randomly allocated into two groups, with each group further divided into two cages housing 5 mice per cage. The mice were provided with unrestricted access to OVA-free food and water throughout the duration of the study. Prior to the commencement of the experiment, all mice underwent a standard rearing protocol for 1 week in order to acclimate environment. The animal experimental protocols were conducted in accordance with the animal research: reporting *in vivo* experiments (ARRIVE) guidelines and the study was reviewed and approved by the Ethics Committee on Animal experiment of Affiliated Hospital of Qingdao University (Number: AHQU-MAL20220729QCY).

### 2.2 Immunization regimen

**Figure 1:**
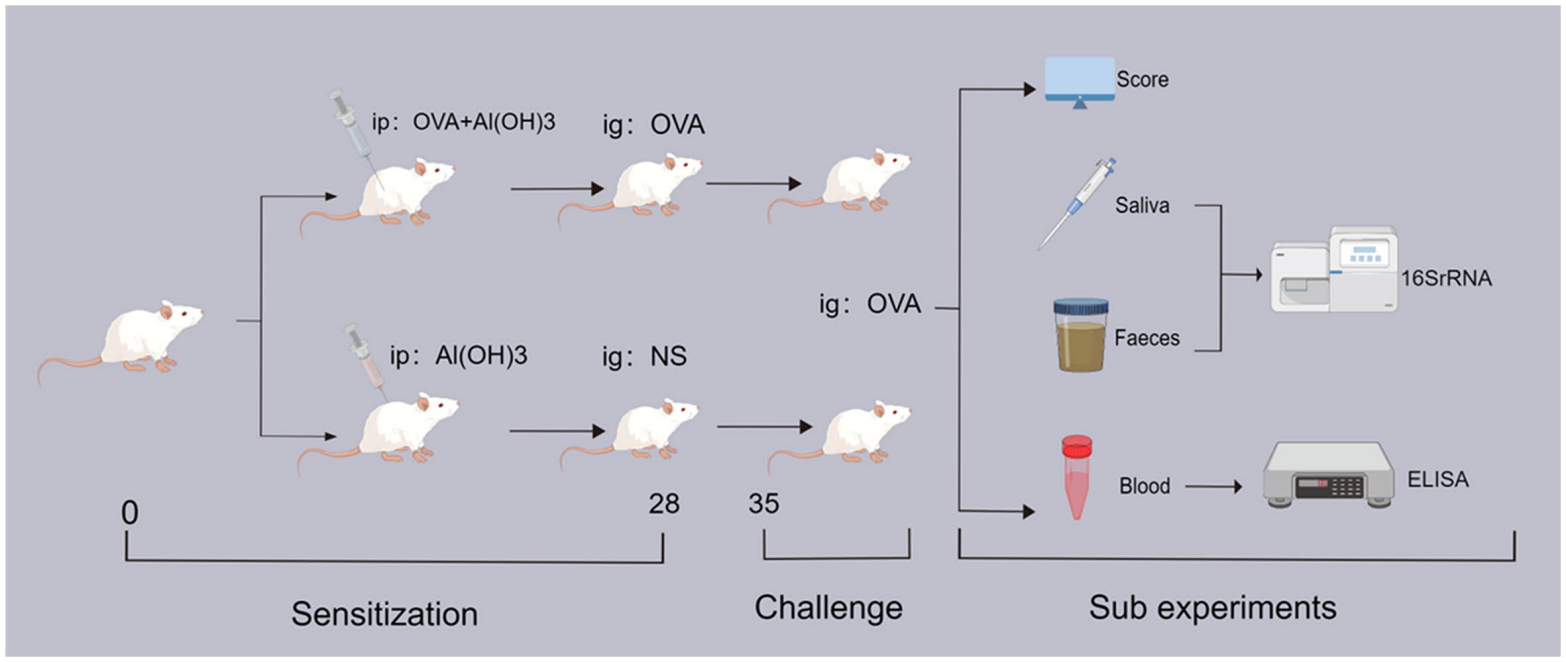
The experimental flow chart. ip: intraperitoneal drug injection; ig: oral gavage medication; OVA: ovalbumin; NS: physiological saline; 16SrRNA: bacterial ribosome sequencing technology; ELISA: enzyme linked immunosorbent assay.

Mouse sensitization was conducted according to the method by Xu et al. (2022), Andreassen et al. (2018), and Diesner et al. (2016) with innovative modifications. Figure 1 provides a schematic representation of the study design. On the initial day, female BALB/c mice in good health were assigned randomly to either the sensitization group (OVA group) and the control group (Con group). The OVA group received 50 μg OVA (Sigma-Aldrich, St. Louis, MO, United States) combined with 1 mg AI(OH)_3_ (Sigma-Aldrich, United States), dissolved in 0.5 mL of normal saline (NS) on day one and again on day seven, via intraperitoneal injection. The control group received 1 mg AI(OH)_3_ dissolved in 0.5 mL of NS, only. Subsequently, the OVA group underwent intensive sensitization through oral administration of 50 μg of OVA dissolved in 0.5 mL of NS, administered one times every 7 days. Conversely, the control group received only 0.5 mL NS. If the experimental animals exhibit an-orexic symptoms, considerable reduction in body weight, or signs of mental and behavioral fatigue during sensitization, they will be excluded from the study or humanely euthanized if necessary.

The mice were weighed on a weekly basis, and all repeated experiments and data measurements were consistently conducted at the same designated time each day. On day 34, all mice were fasted for 2 h, and subsequently, feces and saliva samples were collected using sterile test tubes. On day 35, all mice were orally challenged with 500 μg of OVA (0.5 mL of NS) given after a 6-h fast. The allergic symptoms in mice were eval-uated and statistically assessed within 30–60 min following oral stimulation, subsequent to which blood samples were collected from the mice using serum separator tubes. Finally, after the completion of all experiments, mice were humanely euthanized and colon tissue samples were collected. The total number of mice in each group was as follows: OVA-group (*n* = 10), Con-group (*n* = 10).

### 2.3 Anaphylactic symptom scores and serum IgE and OVA-specific IgE analyzing

To evaluate the intensity of symptoms, a pre-established scoring system for anaphylaxis was utilized (Fu et al., 2017). Clinical anaphylactic symptoms, such as pruritus around the nasal area or periorbital edema, were examined 30 min following oral challenge (Table 1).

**Table 1:** Scoring criteria for clinical anaphylactic symptoms in mice.

Score	Symptom
0	No symptoms
1	Scratching and rubbing around the nose and head
2	Puffiness around the eyes and mouth, diarrhea, pilar erection, reduced activity, and/or decreased activity with increased respiratory rate
3	Wheezing, labored respiration and cyanosis around the mouth and the tail
4	No activity after prodding or tremor and convulsion
5	Death

The retro-orbital venous plexus of mice was utilized for blood collection, followed by centrifugation to obtain serum. Subsequently, the obtained serum was subjected to enzyme-linked immunosorbent assay (ELISA) analysis in order to determine the levels of IgE and sIgE. The levels of IgE and sIgE in serum were quantified using enzyme immunoassay kits (Elabscience, Wuhan, China) following the manufacturer’s instructions. The samples were subjected to analysis with a dilution factor of 1:200.

### 2.4 Histopathological examination

The colonic tissues of the mice were aseptically excised and placed in 4% paraformal-dehyde overnight. Subsequently, they were transferred into PBS solution. The colon was transversely sectioned with a randomized starting point and a cutting interval of 2–3 cm to obtain systematic and uniformly distributed samples. Hematoxylin/eosin (HE) staining was performed on thin sections (3–4 μm in thickness) of the colonic samples that were embedded, aiming to observe inflammatory infiltrates.

### 2.5 Saliva and fecal collection

Prior to the experimental challenge, mouse fecal pellets were collected by employing sterile Eppendorf (EP) tubes, ensuring a minimum of five fecal pellets per mouse were obtained. After sampling was complete, the mice were anesthetized with isoflurane inhalation and placed in a supine position on a 37°C-thermostat pad. The limbs were secured and the mouths were gently opened with sterile metal forceps to facilitate natural saliva secretion, which was subsequently transferred to a sterile centrifuge tube using a micropipette to slowly and gently aspirate the saliva. This process was repeated multiple times until a volume >0.5 mL was obtained. Fecal and saliva samples were immediately flash-frozen using liquid nitrogen upon collection and subsequently transferred to a −80°C freezer within a 2-h timeframe for long-term storage until subsequent analysis.

### 2.6 DNA extraction and PCR amplification

The genomic DNA of microbial communities was obtained from Fecal and Saliva samples using the E.Z.N.A.^®^ soil DNA Kit (Omega Bio-tek, Norcross, GA, United States) following the guidelines provided by the manufacturer. The DNA sample was analyzed using a 1% agarose gel, and the concentration and purity of the DNA were assessed using a NanoDrop 2000 UV–vis spectrophotometer (Thermo Scientific, Wilmington, United States). The bacterial 16S rRNA genes were amplified using the universal bacterial primers 27F (5’-AGRGTTYGATYMTGGCTCAG-3′) and 1492R (5’-RGYTACCTTGTTACGACTT-3′) (Weisburg et al., 1991). Reagents were modified with unique PacBio barcode sequences to differentiate individual samples. The reaction mixture included 4 μL of 5 × FastPfu buffer, 2 μL of 2.5 mM dNTPs, 0.8 μL of forward primer (5 μM), 0.8 μL of reverse primer (5 μM), 0.4 μL of FastPfu DNA Polymerase, and finally, the template DNA with a concentration of 10 ng along with DNase-free water was added to complete the reaction mixture. The PCR amplification protocol involved an initial denaturation step at 95°C for a duration of 3 min. This was followed by a series of 27 cycles, each consisting of denaturation at 95°C for 30 s, annealing at 60°C for another 30 s, and extension at 72°C for a period of 45 s. A final single extension step was performed at 72°C for a total duration of 10 min before concluding the process by cooling to 4°C (ABI GeneAmp^®^9,700 PCR thermocycler, CA, United States). The PCR reactions were conducted in triplicate. Following electrophoresis, the PCR products underwent purification utilizing AMPure^®^ PB beads (Pacifc Biosciences, CA, United States) and quantification was performed using the Quantus™ Fluorometer (Promega, WI, United States).

### 2.7 Sequencing library construction

The DNA library was constructed using the SMRTbell^®^ Express Template Prep Kit 2.0 (Pacifc Biosciences, CA, United States) following PacBio’s instructions. Purified SMRTbell libraries were sequenced on the Pacbio Sequel II System (Pacifc Biosciences, CA, United States) by Majorbio Bio-Pharm Technology Co. Ltd. (Shanghai, China).

### 2.8 Data processing and statistical analysis of microbiota data

The PacBio raw reads were subjected to processing using the SMRTLink analysis software (version 8.0) in order to obtain demultiplexed circular consensus sequence (CCS) reads that met a minimum threshold of three full passes and 99% sequence accuracy. CCS reads are subjected to barcode identification and length filtration, ensuring the retention of sequences ranging from 1,000 to 1,800 base pairs in length.

The optimized-CCS reads were clustered into operational taxonomic units (OTUs) using UPARSE 7.1 (Edgar, 2013) at a sequence similarity level of 97%. In order to mitigate the impact of sequencing depth on alpha and beta diversity analysis, we standardized the number of 16S rRNA gene sequences per sample to 6,000. The representative sequence for each OTU was taxonomically classified using RDP Classifier version 2.2 (Wang et al., 2007) against the 16S rRNA gene database (e.g., Silva v138) with a confidence threshold of 0.7. Prediction of metagenomic function based on OTU representative sequences was performed using PICRUSt2 (Phylogenetic Investigation of Communities by Reconstruction of Unobserved States) (Douglas et al., 2020) utilizing microbial functional gene mapping data from the Kyoto Encyclopedia of Genes and Genomes (KEGG).^1^ The oral and gut microbiota were subjected to bioinformatic analysis using the Majorbio Cloud platform.^2^ The rarefaction curves and alpha diversity indices were computed using Mothur v1.30.1 (Schloss et al., 2009). Principal coordinate analysis (PCoA) based on unweighted_UniFrac dissimilarity, as implemented in the Vegan v2.5–3 package, was employed to assess the similarity among microbial communities across different samples.

### 2.9 Linear discriminant analysis effect size analysis and random forest classifier

The Linear discriminant analysis effect size (LEfSe) tool (Segata et al., 2011)^3^ was utilized to detect bacterial species that exhibited significant abundance differences across various groups (LDA score > 2, *p* < 0.05). Subsequently, the randomForest package (version 4.6) in R was employed to construct a random forest model for predictive analysis of biomarkers in distinct species.

### 2.10 Statistical analysis

The results were reported as mean ± standard deviation (SD). Statistical analysis was conducted using GraphPad Prism 9.4.0 (GraphPad Software, California, United States), which included the *t*-test, Spearman’s correlation analysis, and Pearson’s correlation analysis. Statistical significance was defined as a *p* < 0.05.

## 3 Results

### 3.1 Evaluation of body weight, clinical manifestations, and plasma antibody levels in BALB/c mice

**Figure 2:**
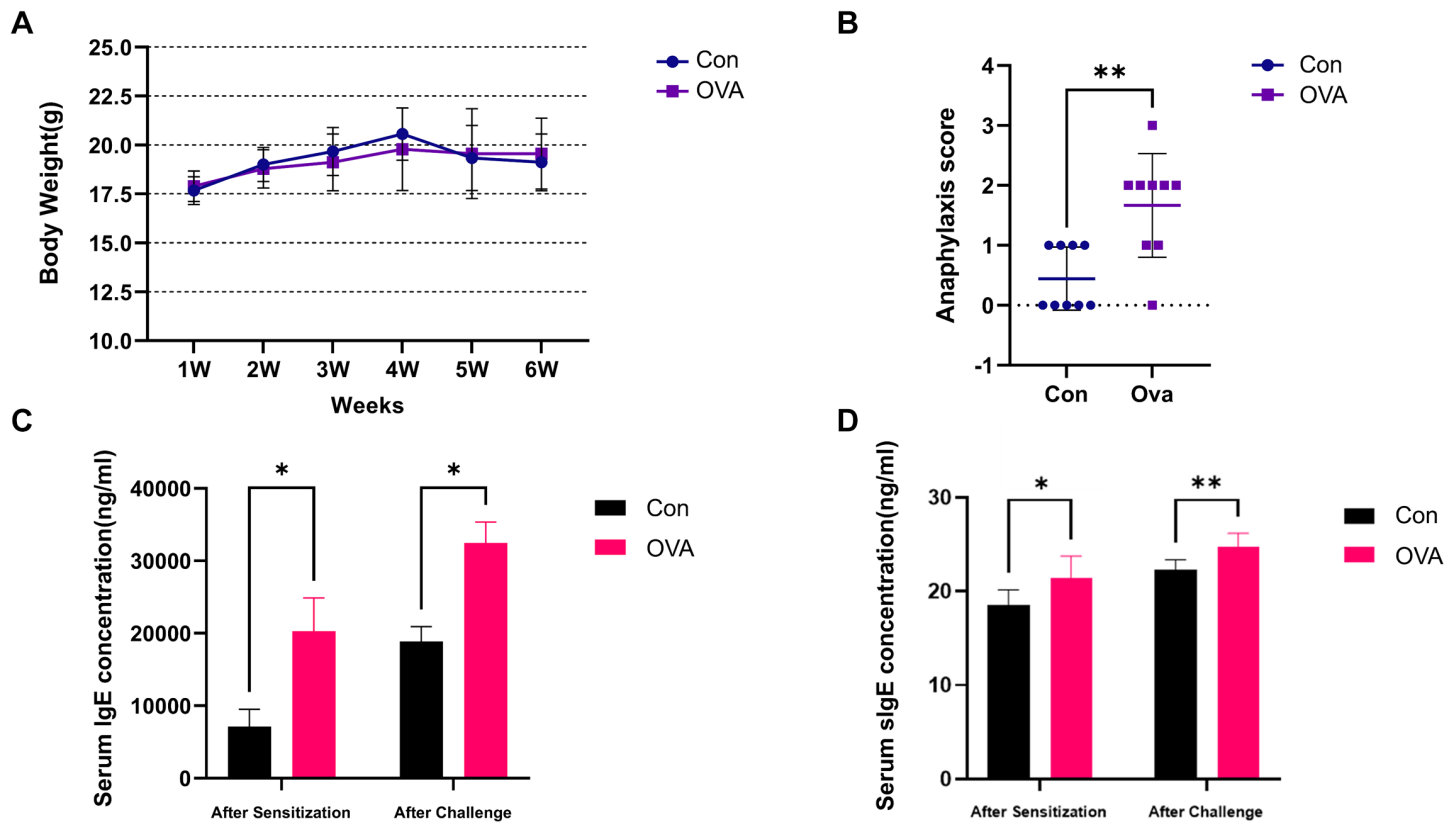
Exposure to OVA leads to enhanced allergic immune responses in BALB/c mice. **(A)** Weekly weight change analysis of BALB/c mice in different groups. **(B)** Anaphylaxis symptom score in different groups. **(C)** ELISA measured IgE levels in the serum. **(D)** OVA-specific IgE level in the serum measured by ELISA. Data are presented as mean ± SEM. Statistical significance was determined by two-sided Student’s *t*-test or one-way ANOVA, ^*^p < 0.05, ^**^p < 0.005.

The results of weekly body weight changes, anaphylactic symptom scores and serum concentrations of IgE as well as sIgE in mice have been presented in Figure 2. The OVA group of mice exhibited a reduced weekly body weight gain compared to the Con group (Figure 2A). And OVA group exhibited significantly higher anaphylactic symptom scores, indicating the successful establishment of FA model (Figure 2B). Apart from the clinical symptoms, the measurement of specific antibodies is considered a reliable approach for assessing allergen sensitization. Consistent with the observed allergic symptoms, the serum levels of sIgE and total IgE were significantly elevated in the sensitized groups (*p* < 0.05), thereby providing robust serological evidence for the FA model (Figures 2C,D).

### 3.2 Effect of ovalbumin on allergic inflammation of the colon in BALB/c mice

**Figure 3:**
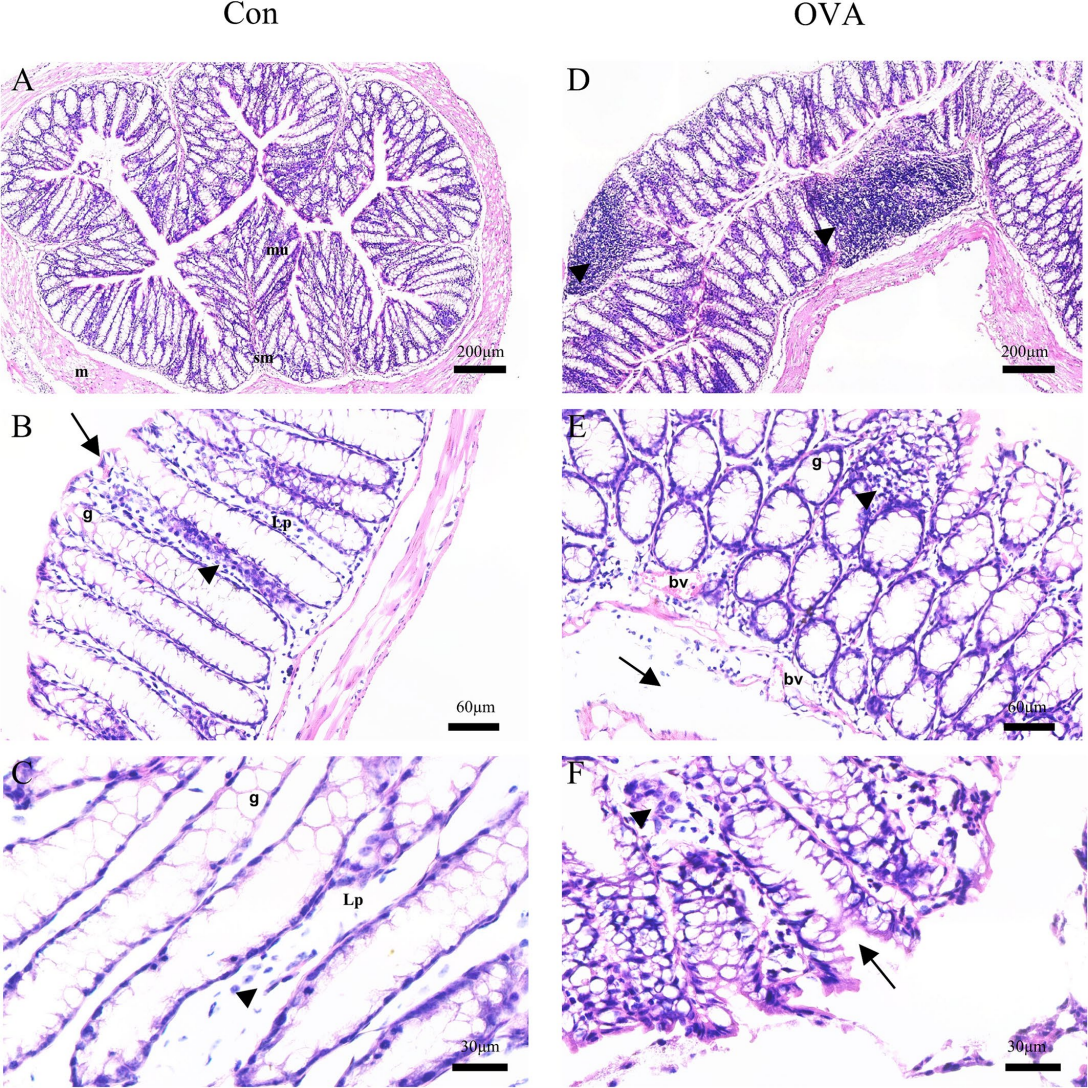
H&E-stained sections of the colon of BALB/c mice. Control group: **(A)** normal arrangement of the lining layers; mucosa (mu), submucosa (sm) and musculosa (m) (20×). **(B)** Apical parts of the mucosa: absorptive columnar cells (arrows), numerous goblet cells (g), thin lamina propria (Lp), and few lymphocytes (arrowheads) (100×). **(C)** Apical parts of the mucosa: numerous goblet cells (g), thin lamina propria (Lp), and few lymphocytes (arrowheads) (200x). OVA group: **(D)** diffuse inflammatory cell infiltrations (arrowheads) (20×). **(E)** Goblet cells (g), congested blood vessels (bv), submucosal edema (arrows), and many inflammatory cell infiltrations (arrowheads) (100×). **(F)** Abnormal crypts (arrows), some inflammatory cells (arrowheads) (400×).

Analysis of H&E-stained colon sections from the control group demonstrated a typical organization of lining layers, including the musculosa, submucosa, and mucosa. The crypts were lined with columnar absorptive cells that were characterized by the presence of acidophilic cytoplasm and oval basal nuclei. Additionally, an abundance of goblet cells with vacuolated cytoplasm and basal nuclei were found, as well as a few lymphocytes (Figures 3A–C). In contrast, sections from the OVA group demonstrated an abnormal structure of the colon. There were noticeably fewer crypts and a greater infiltration of inflammatory cells. The presence of submucosal edema, separation of muscle fibers, and congested blood vessels was observed in addition (Figures 3D–F).

### 3.3 Differential analysis of gut and oral microbiota diversity in different groups of BALB/c mice

**Figure 4:**
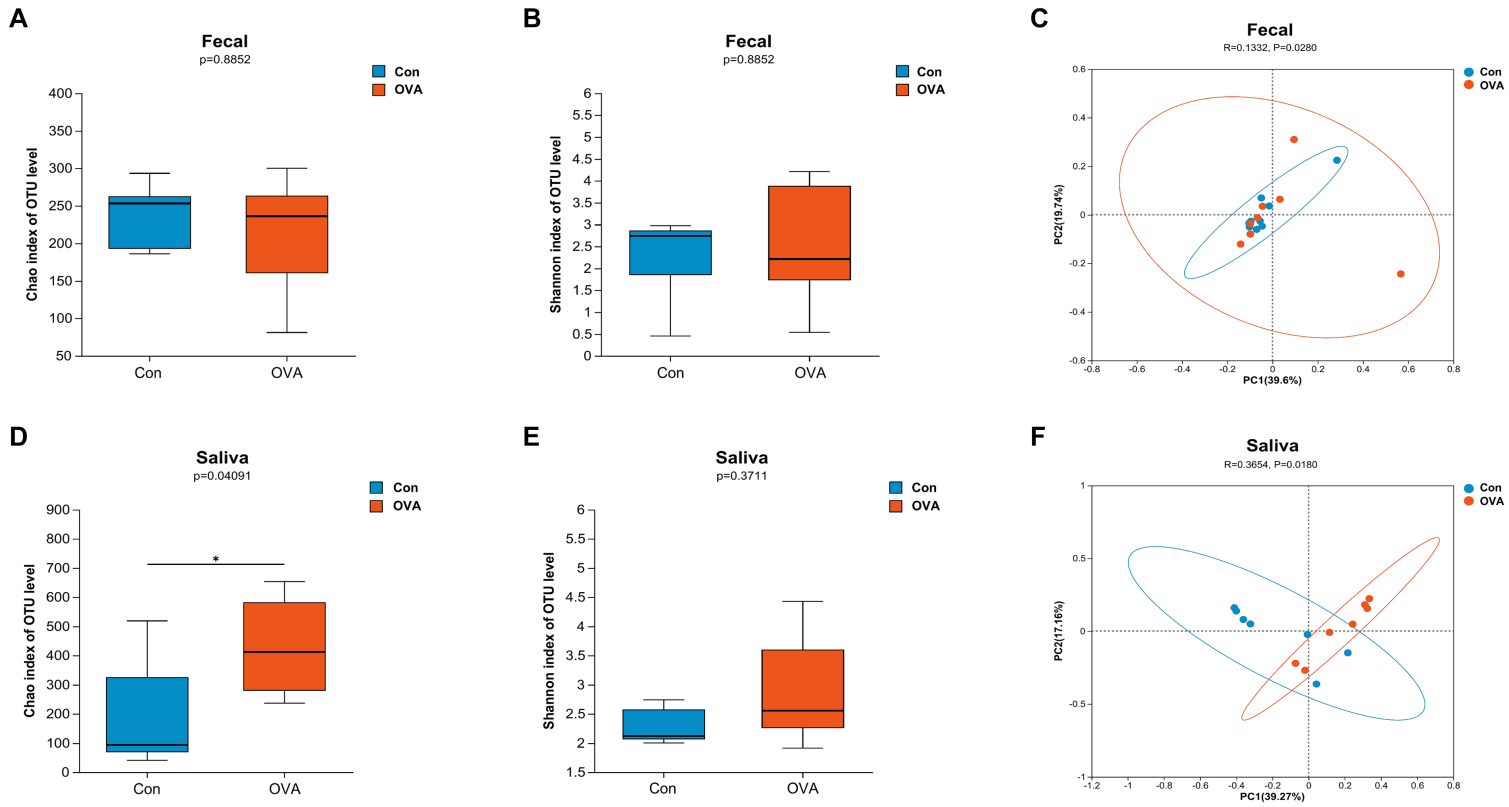
Distinct gut and oral microbiota in BALB/c mice with control group versus OVA group. α-diversity: **(A)** Chao1 index (gut microbiota). **(B)** Shannon index (gut microbiota). **(D)** Chao1 index (oral microbiota). **(E)** Shannon index (oral microbiota). Data are expressed as a two-sided Wilcoxon rank sum test determined means ± SEM and statistical significance, ^*^p < 0.05. β-diversity: **(C)** PCoA of gut microbiota composition in Con and OVA group. **(F)** PCoA of oral microbiota composition in Con and OVA group. PCoA is based on unweight_unifrac distance. ANOSIM and 999 times permutation tests were utilized.

In the present study, we conducted an analysis of the microbiota composition in control and immunized animals by evaluating the samples of feces and oral saliva. To quantify the richness and diversity of the microbiota, we utilized Chao1 and Shannon indexes, respectively. Meanwhile, we employed the unweighted_UniFrac distance algorithm with a 95% confidence level to analyze the beta diversity. The gut microbiota diversity did not exhibit any significant disparities between the OVA group and the control group (*p* Chao1 and *p* Shannon = 0.8852) (Figures 4A,B). The results of beta diversity analysis are illustrated in Figures 4C,F. PCoA revealed a significant difference in the gut microbiota between the OVA and control group (*r* = 0.1332, *p* = 0.028). In addition, PCo1 and PCo2 components contributed 39.6 and 19.7%, respectively, to the overall variation (Figure 4C). However, the OVA group exhibited a significant increase in species richness of oral microbiota compared to the control group (*p* Chao1 = 0.0409) (Figure 4D), indicating a pronounced enhancement in oral microbiota diversity within the OVA group. Furthermore, although the difference did not reach statistical significance, the Shannon index of the oral microbiota exhibited an increase in the OVA group compared to the control group (*p* Shannon = 0.3711) (Figure 4E).

Similarly, a significant difference was observed in the oral microbiota between the two groups (*r* = 0.3654, *p* = 0.018). The PCo1 and PCo2 components contributed 39.27 and 17.16%, respectively, to the overall variation (Figure 4F). Our findings suggest that OVA stimulation significantly influenced the microbial community structures in both the gut and oral regions, as compared to the control group.

### 3.4 The structural composition of gut and oral microbiota in different groups

**Figure 5:**
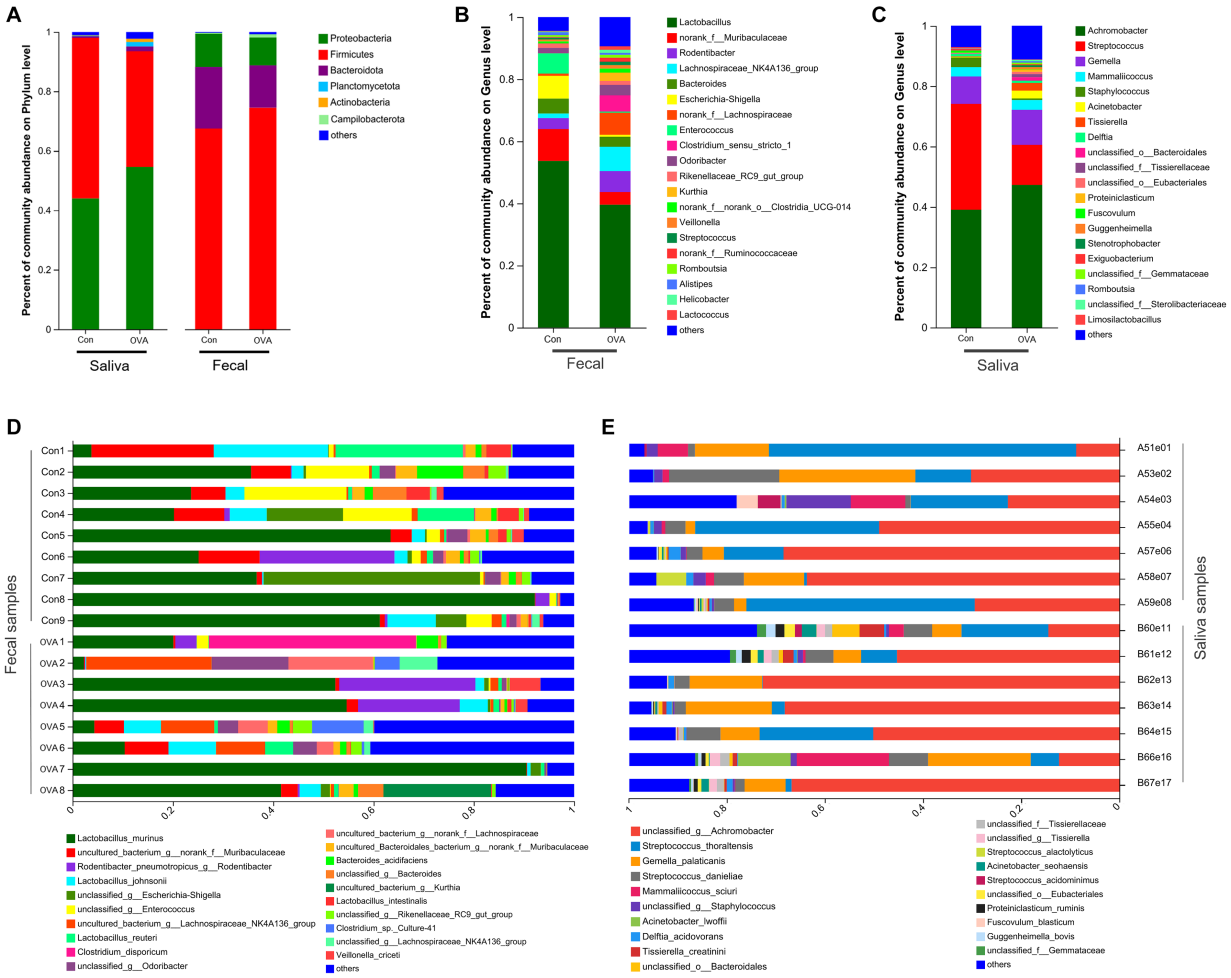
The structural composition of the oral and gut microbiota in BALB/c mice with OVA versus the control group. **(A)** Comparison of relative taxa abundance between OVA and control group at phylum level. **(B)** Comparison of relative taxa abundance between OVA and control group at genus level in fecal samples. **(C)** Comparison of relative taxa abundance between OVA and control group at genus level in saliva samples. **(D)** Bar plot of percentage abundance at the species level in every fecal sample. **(E)** Bar plot of percentage abundance at the species level in every saliva sample.

Figure 5 illustrates the comprehensive taxonomic distribution of microbial communities across different hierarchical levels, including phylum, genus, and species. At the phylum level (Figure 5A), the most abundant groups in the fecal sample were, *Firmicutes* (74.54 and 67.54% of total bacteria in the OVA and control group, respectively), *Bacteroidetes* (14.23% in the OVA group and 20.64% in the control group), *Proteobacteria* (9.35% in the OVA group and 11.19% in the control group), and *Campilobacterota* (1.04% in the OVA group and 0.32% in the control group). The combined abundance of these four dominant phyla constituted over 99% of the bacterial population in both groups. In contrast to the control group, the OVA group demonstrated higher number of *Firmicutes* and *Campilobacterota*, and less *Bacteroidetes* and *Proteobacteria*. The saliva samples contained five dominant phyla (with a relative abundance larger than 1%) namely: *Proteobacteria* (54.59% in the OVA group and 44.03% in the control group), *Firmicutes* (38.92% in the OVA group and 53.95% in the control group), *Bacteroidetes* (1.61% in the OVA group and 0.57% in the control group), *Planctomycetota* (1.47% in the OVA group and 0.23% in the control group), and *Actinobacteria* (1.08% in the OVA group and 0.14% in the control group) (Figure 5A). The aforementioned phyla did not exhibit any discernible differences between the two groups; however, there were significant variations in the abundances of *Acidobacteria*, *Chloroflexi*, *Nitrospirae*, and *Ignavibacteriae* (see Supplementary Table S1).

At the genus level, there were 20 dominant genera identified in the fecal samples; *Lactobacillus* (39.58% in the OVA group and 53.67% in the control group) accounted for the highest proportion, followed by *norank_f__Muribaculaceae* (4.07% in the OVA group and 10.27% in the control group) and *Rodentibacter* (6.72% in the OVA group and 3.56% in the control group) (Figure 5B). Additionally, the saliva samples contained 20 dominant genera, with *Achromobacter* (47.28% in the OVA group and 39.06% in the control group), *Streptococcus* (13.30% in the OVA group and 35.05% in the control group), and *Gemella* (11.60% in the OVA group and 9.14% in the control group) being identified as the three most abundant genera (Figure 5C). Finally, Figures 5D,E portrays the overall taxonomic distribution of microorganisms found in samples collected from feces and saliva at the species levels. (There were 20 dominant species found in both the fecal and saliva samples).

### 3.5 Potentially dangerous microbiota biomarkers identification between OVA and control group and random forest classifier establishment

**Figure 6:**
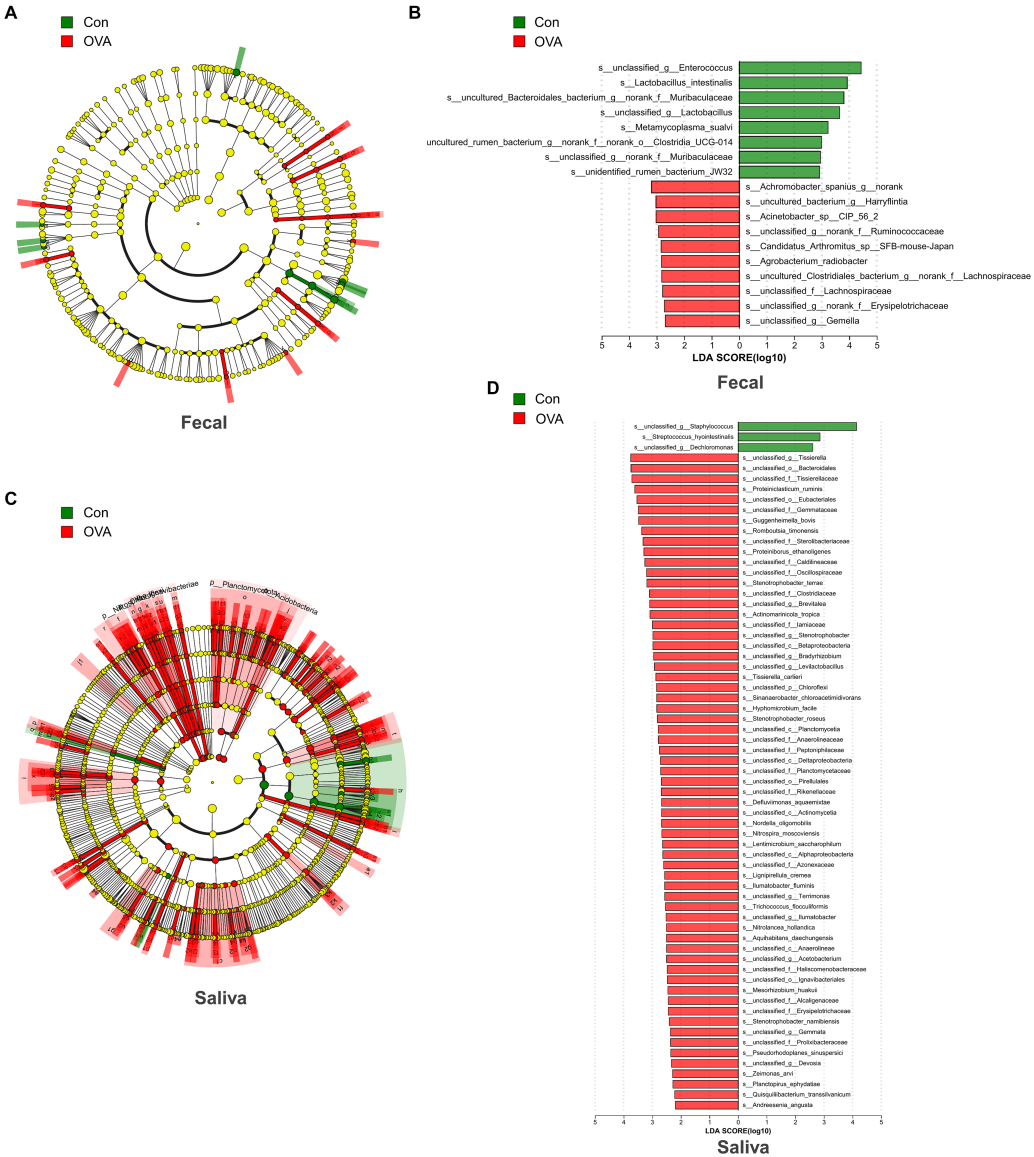
Bacterial difference identification in gut and oral microbiota in OVA group and control group. **(A)** The taxonomic cladogram displayed the enriched taxa in OVA and control gut microbiota. **(B)** Histogram of the LDA scores in gut microbiota between OVA and control group (LDA score  >  2). **(C)** The taxonomic cladogram displayed the enriched taxa in OVA and control oral microbiota. Each node represents a specific taxonomic type (yellow: non-significant; green: control group; and red: OVA group). The diameter of each circle is proportional to the relative abundance of the taxon. (D) Histogram of the LDA scores in oral microbiota between OVA and control group (LDA score > 2).

In order to identify distinct bacterial taxon biomarkers between mice in the OVA group and the control group, we employed LEfSe to detect species exhibiting significant differences in abundance between these two groups. The complete range of microbiota structures, ranging from the phylum to species levels, was depicted using a cladogram (Figures 6A,C). The microbiota exhibiting significant differences in the gut and oral cavity between the two groups were identified based on LDA scores > 2 (Figures 6B,D). Interestingly, there were 18 specific microbes identified in fecal samples, displaying significant variations in their relative abundances between the two groups. In particular, the control group had a higher abundance of *s_unclassified_g__Enterococcus* (*p* = 0.0021), *s_Lactobacillus_intestinalis* (*p* = 0.0343), *s_uncultured_Bacteroidales_bacterium_g__norank_f__Muribaculaceae* (*p* = 0.0433), *s_unclassified_g__Lactobacillus* (*p* = 0.0103) and *s_Metamycoplasma_sualvi* (*p* = 0.0269) etc., and the OVA group had a higher abundance of *s_Achromobacter_spanius_g__norank* (*p* = 0.0072), *s_uncultured_bacterium_g__Harryflintia* (*p* = 0.0200), *s_Acinetobacter_sp__CIP_56_2* (*p* = 0.0072), *s_unclassified_g__norank_f__Ruminococcaceae* (*p* = 0.0374) and *s_Candidatus_Arthromitus_sp__SFB-mouse-Japan* (*p* = 0.0092) etc. (Figure 6B; see Supplementary Table S2).

In the saliva samples, a total of 66 specific microbes at the species level exhibited notable differences in their relative abundances between the two groups. The control group was significantly enriched with three strains of bacteria specifically, *s_unclassified_g__Staphylococcus* (*p* = 0.0127), *s_Streptococcus_hyointestinalis* (*p* = 0.0344), and *s_unclassified_g__Dechloromonas* (*p* = 0.0222) respectively. In contrast, the OVA group was significantly enriched with 63 strains, including mainly *s_unclassified_g__Tissierella* (*p* = 0.0311), *s_unclassified_o__Bacteroidales* (*p* = 0.0176), *s_unclassified_f__Tissierellaceae* (*p* = 0.0176), *s_Proteiniclasticum_ruminis* (*p* = 0.0167) and *s_unclassified_o__Eubacteriales* (*p* = 0.0168), etc. (Figure 6D; see Supplementary Table S3). Next, we established a random forest classifier using these biomarkers to predict disease outcomes. The random forest classifier achieved the best accuracy (94.12%) in predicting the outcome of FA using three gut biomarkers, which were *s__unclassified_g__Gemella*, *s__uncultured_Clostridiales_bacterium_g__norank_f__Lachnospiraceae* and *s__unclassified_f__Lachnospiraceae* (see Supplementary Figure S1A). For the best classifier to predict the outcome of FA, the included oral biomarkers were three, and the accuracy was 100%, which were *s__Proteiniborus_ethanoligenes*, *s__unclassified_o__Ignavibacteriales* and *s__unclassified_c__Betaproteobacteria* (see Supplementary Figure S1B).

### 3.6 Kyoto encyclopedia of genes and genomes pathway analysis

**Figure 7:**
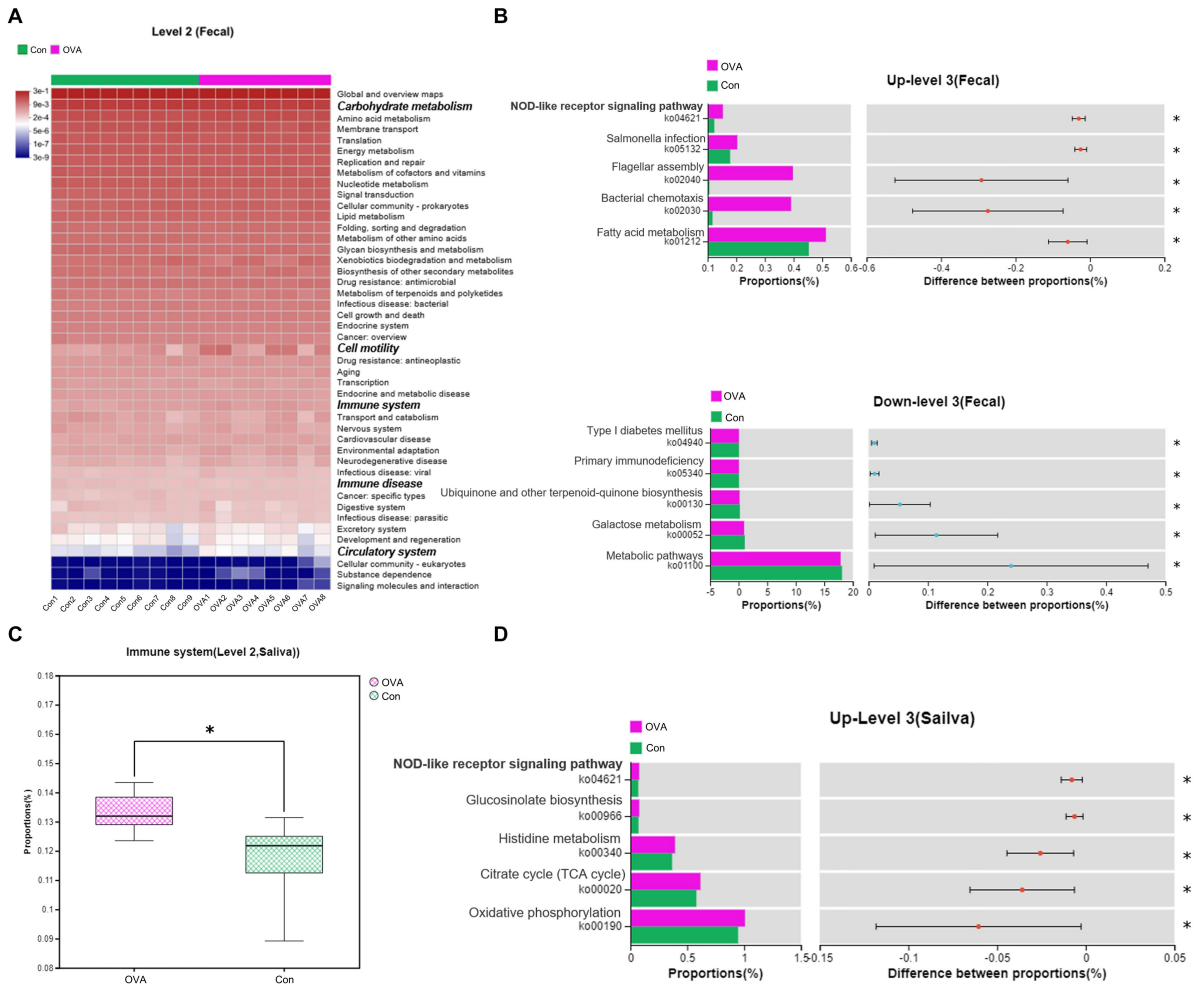
Changes in the predicted intestinal and oral flora function in OVA-allergy mice. Fecal: **(A)** Heat map of Pearson’s hierarchical clustering obtained from the selected pathways (level 2). The italicized and bolded text on the right side shows the pathways that have changed significantly at Level 2. **(B)** The relative abundance comparisons of these pathways (level 3) between the control group and the OVA group. Above is the pathway to upward mobility in the OVA group. Below is the pathway down in the OVA group. Abundance levels are all in the top five. Saliva: **(C)** The relative abundance comparisons of these pathways (level 2) between the control group and OVA group. **(D)** The relative abundance comparisons of these pathways (level 3) between the control group and the OVA group. *t*-test at the 95% level, ^*^p < 0.05.

To investigate the impact of altered gut and oral microbiota on the metabolism in OVA-sensitive mice, we conducted a comparative analysis of the sequencing results with respect to the KEGG database to identify the key pathways. We found significant alterations in the functional abundance of level 2 and level 3 pathways, as per the KEGG classification, when analyzing fecal samples from OVA group. As depicted in Figure 7A, a significant alteration in five level 2 pathways (*p* < 0.05) was observed in the OVA group compared to the control group, encompassing carbohydrate metabolism, cell motility, immune system, immune disease, and circulatory system pathways. We further analyzed these level 2 pathways and identified the significantly altered level 3 pathways among them, as shown in Figure 7B, the pathways that were significantly up-regulated in the OVA group consisted mainly of ko01212, ko02030, ko02040, ko05132, and ko04621 (11 pathways in total), and down-regulated were mainly of ko01100, ko00052, ko00130, ko05340, and ko04940 (eight pathways in total) (see Supplementary Table S4). Moreover, analysis of data from saliva samples from OVA group indicated that only one level 2 pathway, namely the immune system, was significantly modified in the OVA group compared to the control group. Remark-ably, this pathway was upregulated in the OVA group, as depicted in Figure 7C. After con-ducting a meticulous examination, we further scrutinized this level 2 pathway and identified the significantly altered level 3 pathways among them. The pathways that were significantly up-regulated in the OVA group consisted mainly of ko00190, ko00020, ko00340, ko00966, and ko04621 (16 pathways in total) (see Supplementary Table S5). Notably, when performing combined analyses of fecal and saliva samples, we noticed that the immune system pathway was the only level 2 pathway that was consistently affected. Furthermore, the ko4621 (NOD-like receptor signaling pathway) emerged as the sole level 3 pathway that showed simultaneous alterations, both of which were upregulated in OVA allergic mice (Figures 7B,D).

## 4 Discussion

Currently, there is a significant and continuous increase in FA (Wood, 2016; Goldberg et al., 2020). However, the specific reason behind this rise remains largely undefined. Recent evidence has highlighted the role of intestinal bacteria in the prevention or treatment of FA, indicating that microbial dysbiosis during early stages of life is crucial in the development of FA (Prince et al., 2015). Furthermore, certain investigations have revealed a strong association between oral microecology and the development of FA (Ho et al., 2021).

In our study, we utilized BALB/c female mice and employed intraperitoneal injection combined with oral gavage administration of OVA to induce OVA sensitivity in these mice. The administration of this treatment significantly impacted the composition of both fecal gut microbiota and salivary oral microbiota. The extent of the allergic response in the mice was accurately represented by the allergic symptoms they experienced, the levels of sIgE and total IgE and the pathological changes observed in their intestinal tissues. For instance, a study using a mouse model of FA to shrimp tropomyosin (ST) revealed that mice sensitized to ST exhibited elevated levels of ST-specific IgE and immunoglobulin G1 (sIgG1) compared to the control group, as well as displaying symptoms of anaphylaxis, including reduced and/or decreased activity accompanied by increased respiratory rates, wheezing, and labored respiration (Fu et al., 2017). Wang et al. (2024) orally administered egg allergen OVA to sensitized BALB/c mice in order to accurately replicate key aspects of human FA. This included the development of severe allergic diarrhea, infiltration of mast cells, and elevated levels of serum IgE, mMCPT-1, and Th2 cell signature cytokines such as IL-4, IL-5, and IL-13 (Wang et al., 2024). In the present study, within 1 h, the administration of OVA through oral gavage resulted in the occurrence of moderate allergic manifestations such as piloerection and rapid breathing or even cyanosis around the mouth and the tail. Additionally, there was a significant increase in the levels of serum OVA sIgE and IgE, confirming the occurrence of an allergic response in the mice that were exposed to OVA. Food allergy is characterized not only by clinical symptoms and abnormalities in specific immune-related proteins but also by the presence of inflammation in intestinal tissues. It is well known the gastrointestinal tract serves as a primary gateway for the entry of food allergens and the intestinal epithelium, acts as an important barrier in promoting tolerance to orally ingested FA (Nowak-Wegrzyn et al., 2017). The presence of local pro-inflammatory signals can contribute to allergic responses by causing damage to the epithelial barrier function and promoting an enhanced intake of food antigens and allergens (Diesner et al., 2016). In our model, we observed a large number of inflammatory cells, submucosal edema, disruption of the muscular layer, and congestion of the various blood vessels in the sensitized group, which is consistent with the findings of Belkaaloul et al. (2015), Diesner et al. (2016), and Brandtzaeg (2010). These findings underscore the crucial role of intestinal inflammation in the development of FA.

The administration of OVA through gavage can potentially modulate the sensitivity of mice to OVA by perturbing the intestinal microbiota. Concurrently, the composition of oral saliva microbiota in mice also undergoes alterations, but the exact cause effect relationship is not yet known. In our study, we utilized full-length 16S rRNA gene sequencing to investigate the gut and oral microbiota, thereby revealing distinct compositions in OVA-allergic mice compared to their healthy counterparts. Alpha diversity analysis, which assesses species richness, diversity, and evenness, was used. In particular, the Chao1 and Shannon indexes were employed to measure the species richness and diversity, respectively. For instance, Lee et al. (2021) discovered that children with IgE-mediated FA exhibited higher species richness (Chao1 index) in their gut microbiota in comparison to the healthy children, and Fazlollahi et al. (2018) observed increased bacterial diversity (Chao 1 and Shannon indices) in the fecal samples of children affected with egg allergies. Despite the abundance of research on the relationships between different food allergy and the gut microbiota, the literature on the oral microbiota remains limited, particularly in animal models. Dzidic et al. (2018) analyzed the saliva from children with allergic asthma in comparison with saliva from healthy children and found that the bacterial diversity in the saliva of 7-year-olds with allergic diseases such as asthma was significantly reduced. In addition, Ho et al. (2021) reported similar findings in their investigation of saliva samples from individuals with peanut allergies, while Zhang et al. (2022) observed variations in oral microbiota diversity among patients with different levels of peanut allergy, with children with low-threshold peanut allergy showing higher α diversities in the oral microbiota than children with high-threshold peanut allergy. In the present study, while no significant difference was seen between the α diversities of the two groups, mice with OVA allergy displayed higher α diversities in the oral microbiota than those in the control group. The above evidence indicates that the diversity of species in the gut and oral cavity of food allergic individuals is not limited to one pattern of variation and that even the microbiota diversity among food allergic individuals leads to different patterns of variation depending on the specific type of FA (Goldberg et al., 2020). There is a complex connection between microbial diversity and specific diseases, making it difficult to generalize the impact of the microbiome on overall health and disease solely based on alpha diversity.

However, the changes in community composition were more noteworthy than the changes in microbiota diversity. Several studies have suggested that significant alterations in the abundance of distinct flora within the gut and oral cavity could be observed in individuals with allergies, and these specific florae could serve as valuable targets for disease diagnosis, prevention, and treatment. For instance, Xu et al. (2022) demonstrated that mice affected with OVA-induced allergic diarrhea displayed a significant reduction in the relative abundance of *Rikenellaceae*, *Muribaculaceae*, *Erysipelotrichaceae*, and *Christensenellaceae* families in their gut microbiota, along with a significant increase in the abundance of *Lactobacillaceae* family. Duan et al. (2023) further reported that OVA-induced mice displayed specific gut microbial characteristics, including an increase in *Firmicutes* abundance and a decrease in *Bacteroidetes* abundance. Notably, this alteration was reversed following the oral administration of *L. plantarum* JC7. *L. plantarum* JC7 effectively decreased levels of OVA-specific IgE while also restoring the balance between Th1/Th2 and Treg/Th17 by enhancing the secretion of IL-10 and IFN-γ and suppressing the secretion of IL-4 and Th17. Additionally, *L. plantarum* JC7 modified the disrupted intestinal microbiota, effectively preventing FA.

In our study, we noted a significant decrease in the abundance of *s_Lactobacillus_intestinalis* in the fecal samples of OVA-sensitive mice. Moreover, we noticed a substantial decline in taxa belonging to the orders *Clostridia_UCG-014* and *Clostridia_vadinBB60_group*, as well as taxa from the *Enterococcus* genera (Figure 7B, Supplementary Table S4). *S_ Lactobacillus_intestinalis* belongs to the genus *Lactobacillus*, which, together with *Enterococcus*, belongs to the order *Lactobacillales*, while *Clostridia_UCG-014* and *Clostridia_vadinBB60_group* belong to the class *Clostridia*. These resilient species possess the ability to survive in important biological niches, such as the human gastrointestinal tract, and are of immense significance in the fields of economics and medicine (García-Solache and Rice, 2019). For instance, Schei et al. (2021) discovered a significant overrepresentation of multiple *Enterococcus* spp. in healthy children at 2 years old in comparison to the children with eczema. In a study related to the fecal microbiome in food-allergic twins, Bao et al. (2021) observed that taxa from the class *Clostridia* were significantly more enriched in healthy twins. Furthermore, these microorganisms hold the potential to serve as probiotics, offering a viable approach to mitigate the symptoms associated with food allergy. For example, certain strains of *Enterococcus* have exhibited the capability to decrease TH17 activation and ease allergic airway conditions in mouse models (Hanchi et al., 2018; Pascal et al., 2018). Interestingly, the results of a study conducted on mice showed that mice treated with a combination of β-Lg supplemented with *Enterococcus* had substantially less intestinal inflammation compared to those treated with β-Lg alone (Belkaaloul et al., 2015). This is related to the ability of *Enterococcus* to hydrolyze the allergenic substance β-lactoglobulin (β-Lg) present in the milk. *Enterococcus* is not the sole microorganism that can be employed as a probiotic for the treatment of food allergy. Jin et al. (2021) reported that prophylactic supplementation of the cesarean rats with probiotic mixtures containing *Lactobacillus* and *Bifidobacterium rhamnosus* significantly reduced allergic reactions to OVA. Additionally, several clinical trials have demonstrated the efficacy of *Lactobacillus rhamnosus* GG in alleviating allergic symptoms in infants and young children (Berni Canani et al., 2016; Cukrowska et al., 2021). Tian et al. (2022) and Vanderhoof and Young (2003) found that *Lactobacillus* can enhance the intestinal barrier function, thereby reducing the passage of allergens. Additionally, it can inhibit the occurrence of allergic reactions by stimulating the production of regulatory T cells (Treg cells) (Tian et al., 2022). Meanwhile, *Clostridia* is a crucial group of intestinal microbiota that plays an essential role in maintaining the balance of the intestinal microecology. Research has shown that *Clostridia* can stimulate the production of short-chain fatty acids (SCFA), which possess anti-inflammatory properties and can mitigate the occurrence of food allergies by suppressing Th2-type immune responses (Marrs et al., 2021). *Enterococcus* is a prevalent bacterium in the gut and research has indicated that *Enterococcus* can mitigate reactions to food allergy by producing antimicrobial peptides and modulating intestinal immune responses, thereby promoting local immune tolerance (Lin et al., 2024). *Lactobacillus*, *Clostridia* and *Enterococcus* affect food allergies through different mechanisms. These microorganisms may all play a role in the prevention and treatment of food allergy by modulating the intestinal immune system, enhancing intestinal barrier function, and promoting the production of beneficial metabolites. Abdel-Gadir et al. (2019) discovered that probiotics exert their therapeutic effects by stimulating regulatory T (Treg) cells to express the transcription factor ROR-γT in a MyD88-dependent manner. However, this pathway is absent in infants with FA or in mice, leading to ineffective induction of the microbiota. Therefore, commensal bacteria activate the MyD88/ROR-γt pathway in neonatal Treg cells to prevent FA, while ecological dysregulation disrupts this regulatory response and promotes disease.

In our study, we identified eight gut microbial species that were significantly enriched in the healthy control group. Several of these species are associated with various diseases, including type 2 diabetes, Crohn’s disease, and colitis (Wang et al., 2020; Deng et al., 2022; Leibovitzh et al., 2022). However, the association of these bacteria with FA has not been explored until now, making this study the first to shed light on such an association. In summary, these gut microbes have the potential to treat food allergy, which will be the main focus of our subsequent research work.

In contrast, we observed that an increased abundance of 10 specific species characterized the gut microbiome of mice with OVA allergy, such as *s_Agrobacterium_radiobacter* and *s_Acinetobacter_sp__CIP_56_2*, as well as several species not yet categorized into existing species (Figure 6B, Supplementary Table S4). Interestingly, all 10 species were from two phyla, the *Firmicutes* and *Proteobacteria*. Consistent with our findings, a prior study, which analyzed the feces of 141 children with egg allergy, revealed an enrichment of *Firmicutes* in subjects with egg allergy (Fazlollahi et al., 2018). Additionally, Duan et al. (2023) reported that the relative abundance of *Firmicutes* was significantly higher in OVA-sensitized mice in comparison to the healthy controls. This phenomenon can also be observed in certain phyla of *Proteobacteria* (Hufnagl et al., 2020). We further examined the fecal flora in mice from the OVA group and discovered an enrichment of taxa belonging to the families *Ruminococcaceae* and *Lachnospiraceae*. This discovery coincides with a study conducted by Berni Canani et al. (2016), which also reported an increase in these species among individuals who have milk allergies. Noval Rivas et al. (2013) also observed coordinated changes in the abundance of several Bacteriaceae taxa, such as *Lachnospiraceae*, *Lactobacillaceae*, *Rikenellaceae*, and *Porphyromonadaceae* in the intestinal tracts of OVA-sensitized mice. Furthermore, when the microbiota from sensitized mice was transferred into healthy, germ-free mice, it promoted varying degrees of OVA-specific IgE responses and anaphylaxis. Thirdly, for the prediction of FA, we constructed classifiers for these identified biomarkers, finding that the accuracy of three intestinal biomarkers for predicting FA reached 94.12%, indicating their potential for the early prediction of FA in the future.

The correlation between the gut microbial environment and disease has been extensively explored over the years. Recently, however, investigation into the association between the oral microbiological environment and various diseases has emerged as a new and intriguing research area. Specifically, the investigation of oral microbiota in allergic conditions has gained significant interest. For example, in a study examining salivary microecology in a population with peanut allergies, taxa from the orders *Lactobacillales*, *Bacteroidales*, *Bacillales*, and *Streptophyta*, including species from the genera *Prevotella* and *Streptococcus*, were significantly found to be enriched in healthy subjects, whereas an increased abundance of multiple oral *Neisseria* spp. was reported in individuals with peanut allergies (Ho et al., 2021). Another study highlighted that microbiota in oral environments is not only associated with the development of FA but also with reaction thresholds in individuals with challenge-proven peanut allergies. Interestingly, they found that genera such as *Haemophilus* and *Veillonella nakazawae* were more abundant in high-threshold children than in low-threshold children (Zhang et al., 2022). In our study, we found a significant reduction in the abundance of *s_Streptococcus_hyointestinalis* and *s_unclassified_g__Staphylococcus* in saliva samples collected from the mice that were sensitized with OVA (Figure 6D, Supplementary Table S5). These bacterial taxa belong to the genera *Streptococcus* and *Staphylococcus*, respectively. Furthermore, *Streptococcus* is derived from the orders *Lactobacillus*, but *Staphylococcus* is derived from the orders *Bacillus*. Therefore, the findings of the present study are consistent with those of Ho et al. (2021) and Matsui et al. (2019) but demonstrate a higher level of precision primarily attributed to the superior resolution provided by the use of third-generation sequencing for species identification. In addition, we identified a species of the *Dechloromonas* genus that was found to be significantly enriched in our healthy control group; however, its significance remains unclear due to limited investigation on this particular strain. Furthermore, we noticed another intriguing phenomenon whereby 63 species were significantly enriched in the oral environment of OVA-allergic mice, which represents a much larger number than that detected in the intestinal environment (Figure 6D, Supplementary Table S5). It is well-established that there is a significant disparity in the number of microbial species between the oral and intestinal microbiomes. The oral microbiota is relatively simple and consists of tens to hundreds of microbial species, while the gut microbiota is more diverse, containing hundreds to thousands of microbial species (Kil and Swanson, 2011; Dominguez-Bello et al., 2019). However, our study revealed a more substantial and precise number of significantly distinct species identified in the oral environment of the two groups of mice, which were subjected to the same conditions. As a result, it can be inferred that the dysregulation of the oral microbiota in ovalbumin-allergic mice was more significant compared to the gut microflora. We also built classifiers for the above identified oral biomarkers and found that the three oral biomarkers (*s__Proteiniborus_ethanoligenes*, *s__unclassified_o__Ignavibacteriales,* and *s__unclassified_c__Betaproteobacteria*) predicted FA with 100% accuracy, which was higher than the accuracy of the intestinal markers. Hence, these findings suggest that the use of saliva provides a convenient and non-invasive means of analyzing the upstream microbiome and its symbiotic by-products (Malamud, 1992). Moreover, this method is more accurate than fecal analysis in the detection and prediction of FA.

Based on PICRUSt2 analysis, it was predicted that the fecal and salivary microbiomes of mice in the OVA group exhibited upregulation in 11 and 16 pathways, respectively, in comparison to the control group (Supplementary Table S4). These upregulated pathways were mainly concentrated in metabolism, organismal systems, and human diseases (Level 1). Goldberg et al. (2020), in their analysis of gut microbiology in allergic patients, observed significant enrichment of 5 acetate-related KEGG pathways in non-allergic individuals compared to allergic patients, and the functional shifts in the enriched acetate-related pathways were predominantly influenced by *Bacteroides*. Xu et al. (2022) demonstrated an elevation in the metabolic capacity of various amino acids in the gut microbiota of OVA-sensitive mice, whereas the capacity for aminoacyl-tRNA biosynthesis and several carbohydrates (except for pentose and glucuronide) was found to be decreased. In addition, Pérez-Losada et al. (2023) employed 16S rRNA massive parallel sequencing to characterize the oral bacteriome of individuals affected with allergic rhinitis, allergic rhinitis with asthma, and healthy controls. They found that the bacteriomes of allergic rhinitis and allergic rhinitis with asthma patients exhibited substantial upregulation of 15 and 28 metabolic pathways (analyzed with PICRUSt2), which were primarily associated with degradation and biosynthesis processes. Based on above studies, it appears that dysbiosis in either oral or fecal microbiota may disrupt the normal functioning of bacterial communities, which in turn could potentially impact the onset of hypersensitivity (Pérez-Losada et al., 2023). Previous studies have focused solely on the analysis of the microbiota from specific locations, such as feces (Lee et al., 2021; Xu et al., 2022) or saliva (Ho et al., 2021; Zhang et al., 2022; D'Auria et al., 2023). However, in our study, we not only analyzed the gut and oral environments individually but also explored and examined common characteristics shared between the two domains. To the best of our knowledge, as far as we know, there have been no studies conducted to date that utilize a combined analysis of gut and oral microecology to predict the metabolic function within the microbiome of the hypersensitive response. Interestingly, we discovered only one common pathway, namely, the up-regulated NOD-like receptor signaling pathway (at Level 3). The intracellular NOD-like receptor (NLR) family contains more than 20 members in mammals and plays a key role in the recognition of various intracellular ligands (Caruso et al., 2014). The NLRP3 inflammasome complex, consisting of NLRP3, an adaptor molecule called apoptosis-associated speck-like protein containing a caspase recruitment domain (ASC), and pro-caspase 1, is a multiprotein oligomer that acts as a key player in regulating in innate immunity (Johnson et al., 2005). Upon recognition of pathogen-associated molecular patterns (PAMPs) or damage-associated molecular patterns (DAMPs), the inflammasome components assemble, self-oligomerize (Besnard et al., 2011), recruit the cystathionase recruitment domain (CARD) of ASC, and then activate caspase-1 (Liston and Masters, 2017). Activated caspase-1 can, after that, initiate the conversion of pro-IL-1β and pro-IL-18 into their mature active forms (Fernandes-Alnemri et al., 2007). IL-1β is a crucial pro-inflammatory cytokine responsible for the initiation and maintenance of mast cell-induced allergic inflammation (Basu et al., 2015). This, in turn, can trigger the infiltration, differentiation, and activation of Th2 cells, leading to the secretion of Th2 cytokines, recruitment of dendritic cells to lymph nodes, and the exacerbation of Th17 inflammatory reactions (Besnard et al., 2012; Simpson et al., 2014). These effects are strongly associated with the severity of hypersensitivity. Furthermore, IL-18 can activate mast cells (Kim et al., 2014), contribute to Th17 cell differentiation, play a role in Th1 cell polarization, and serve as a potential cofactor in Th2 cell development as well as IgE production by promoting Th2 cytokine production (Kim et al., 2022). Accordingly, when there is an overproduction of the NLRP3 inflammasome or its effect lasts too long, it activates the NLR signaling pathway, resulting in inflammatory tissue damage and the development of different diseases, such as allergies (Ma et al., 2023). For instance, when Soybean-allergic piglets were given Soybean glycinin and β-conglycinin, it was observed that their intestinal tissues exhibited increased levels of NLRP3 inflammasome ASC, caspase-1, IL-1β, and IL-18 expression (Wang et al., 2023). The immune response in piglets was activated by the soybean antigen protein, causing inflammation, oxidative stress, NLRP3 activation, and a significant increase in the release of pro-inflammatory factors (IL-1β and IL-18). Hence, a fresh approach to the NOD-like receptor signaling pathway could lead to innovative strategies for addressing hypersensitivity treatment. In a recent study conducted by Wang et al. (2021), MCC950, a small molecule inhibitor that selectively targets cytokine release, was found to effectively suppress the production of NLRP3 and IL-1β in mice and macrophage cells. This significant inhibition resulted in decreased inflammation in the bronchial region and showed potential in treating OVA-induced asthma. Hence, the research findings indicate that inhibition of the NLR pathway for treating FA shows considerable potential, and our studies and continuous endeavors hold the promise of furnishing a unique approach to the diagnosis and treatment of FA.

## 5 Conclusion

In summary, FA has complex and intricate associations not only with the gut microbiota but also with the oral microbiota. Our findings reveal that mice who were sensitized to OVA exhibited significant changes in the composition and structure of their gut and oral micro-biota. Remarkably, the alterations observed in the oral microbiota of food-allergic mice surpassed those of the gut microbiota in both magnitude and significance. Furthermore, our study identified three strains specific to the gut microbiota and a further three specifics to the oral microbiota, which served as valuable biomarkers for FA screening, shown by their significant diagnostic accuracy. Furthermore, the accuracy of the oral model classifier surpassed that of the gut model, implying that these oral strains may be more precise than the gut strains for diagnosing FA. Finally, it is important to mention that the NLR signaling pathway plays a vital role in the pathogenesis of OVA-induced allergy. In the future, targeting the NLR signaling pathway to suppress food allergy may represent a promising therapeutic strategy. Nevertheless, the study has several limitations. These include incomplete representation of the human subjects by preclinical models and the relatively small sample size used in the experiments. Furthermore, the oral and intestinal ecosystems include not only microbiota but also a plethora of metabolites and immune factors, which require comprehensive analysis to fully understand the mechanisms underlying FA. Hence, our forthcoming research will concentrate on two primary avenues. Firstly, we aim to broaden the sample size by recruiting individuals afflicted with FA and conducting an exhaustive analysis of their oral microecological environment and, secondly, by integrating the NLR signaling pathway with the dis-tinct strains identified in this study, achieving the goal of treating FA in animal models.

## Data Availability

The original contributions presented in the study are included in the article/Supplementary material, further inquiries can be directed to the corresponding authors.

## Footnotes

^1^https://www.genome.jp/kegg/

^2^https://cloud.majorbio.com

^3^http://huttenhower.sph.harvard.edu/LEfSe

## References

[ref1] Abdel-Gadir A. Stephen-Victor E. Gerber G. K. Noval Rivas M. Wang S. Harb H.. (2019). Microbiota therapy acts via a regulatory T cell MyD88/RORγt pathway to suppress food allergy. Nat. Med. 25, 1164–1174. doi: 10.1038/s41591-019-0461-z, PMID: 31235962 PMC6677395

[ref2] Andreassen M. Rudi K. Angell I. L. Dirven H. Nygaard U. C. (2018). Allergen immunization induces major changes in microbiota composition and short-chain fatty acid production in different gut segments in a mouse model of lupine food allergy. Int. Arch. Allergy Immunol. 177, 311–323. doi: 10.1159/000492006, PMID: 30244242

[ref3] Atarashi K. Tanoue T. Shima T. Imaoka A. Kuwahara T. Momose Y.. (2011). Induction of colonic regulatory T cells by indigenous Clostridium species. Science 331, 337–341. doi: 10.1126/science.1198469, PMID: 21205640 PMC3969237

[ref4] Bao R. Hesser L. A. He Z. Zhou X. Nadeau K. C. Nagler C. R. (2021). Fecal microbiome and metabolome differ in healthy and food-allergic twins. J. Clin. Invest. 131, 1–17. doi: 10.1172/jci141935, PMID: 33463536 PMC7810484

[ref5] Basu R. Whitley S. K. Bhaumik S. Zindl C. L. Schoeb T. R. Benveniste E. N.. (2015). IL-1 signaling modulates activation of STAT transcription factors to antagonize retinoic acid signaling and control the TH17 cell-iTreg cell balance. Nat. Immunol. 16, 286–295. doi: 10.1038/ni.3099, PMID: 25642823 PMC4790724

[ref6] Belkaaloul K. Haertlé T. Chobert J. M. Merah R. Taibi K. Saad El Hachemi H. A.. (2015). Protective effect of *Enterococcus faecalis* DAPTO 512 on the intestinal tract and gut mucosa: milk allergy application. Benef. Microbes 6, 679–686. doi: 10.3920/bm2014.0143, PMID: 26192744

[ref7] Ben-Shoshan M. Clarke A. E. (2011). Anaphylaxis: past, present and future. Allergy 66, 1–14. doi: 10.1111/j.1398-9995.2010.02422.x, PMID: 20560905

[ref8] Berni Canani R. De Filippis F. Nocerino R. Paparo L. Di Scala C. Cosenza L.. (2018). Gut microbiota composition and butyrate production in children affected by non-IgE-mediated cow's milk allergy. Sci. Rep. 8:12500. doi: 10.1038/s41598-018-30428-3, PMID: 30131575 PMC6104073

[ref9] Berni Canani R. Sangwan N. Stefka A. T. Nocerino R. Paparo L. Aitoro R.. (2016). *Lactobacillus rhamnosus* GG-supplemented formula expands butyrate-producing bacterial strains in food allergic infants. ISME J. 10, 742–750. doi: 10.1038/ismej.2015.151, PMID: 26394008 PMC4817673

[ref10] Besnard A. G. Guillou N. Tschopp J. Erard F. Couillin I. Iwakura Y.. (2011). NLRP3 inflammasome is required in murine asthma in the absence of aluminum adjuvant. Allergy 66, 1047–1057. doi: 10.1111/j.1398-9995.2011.02586.x 21443539

[ref11] Besnard A. G. Togbe D. Couillin I. Tan Z. Zheng S. G. Erard F.. (2012). Inflammasome-IL-1-Th17 response in allergic lung inflammation. J. Mol. Cell Biol. 4, 3–10. doi: 10.1093/jmcb/mjr042, PMID: 22147847

[ref12] Brandtzaeg P. (2010). Update on mucosal immunoglobulin A in gastrointestinal disease. Curr. Opin. Gastroenterol. 26, 554–563. doi: 10.1097/MOG.0b013e32833dccf8, PMID: 20693891

[ref13] Bunyavanich S. Berin M. C. (2019). Food allergy and the microbiome: current understandings and future directions. J. Allergy Clin. Immunol. 144, 1468–1477. doi: 10.1016/j.jaci.2019.10.019, PMID: 31812181 PMC6905201

[ref14] Bunyavanich S. Shen N. Grishin A. Wood R. Burks W. Dawson P.. (2016). Early-life gut microbiome composition and milk allergy resolution. J. Allergy Clin. Immunol. 138, 1122–1130. doi: 10.1016/j.jaci.2016.03.041, PMID: 27292825 PMC5056801

[ref15] Caruso R. Warner N. Inohara N. Núñez G. (2014). NOD1 and NOD2: signaling, host defense, and inflammatory disease. Immunity 41, 898–908. doi: 10.1016/j.immuni.2014.12.010, PMID: 25526305 PMC4272446

[ref16] Consortium HMP (2012). Structure, function and diversity of the healthy human microbiome. Nature 486, 207–214. doi: 10.1038/nature11234, PMID: 22699609 PMC3564958

[ref17] Cukrowska B. Ceregra A. Maciorkowska E. Surowska B. Zegadło-Mylik M. A. Konopka E.. (2021). The effectiveness of probiotic Lactobacillus rhamnosus and *Lactobacillus casei* strains in children with atopic dermatitis and Cow's Milk protein allergy: a multicenter, randomized, double blind, placebo controlled study. Nutrients 13:1169. doi: 10.3390/nu13041169, PMID: 33916192 PMC8066586

[ref18] D'Auria E. Cattaneo C. Panelli S. Pozzi C. Acunzo M. Papaleo S.. (2023). Alteration of taste perception, food neophobia and oral microbiota composition in children with food allergy. Sci. Rep. 13:7010. doi: 10.1038/s41598-023-34113-y, PMID: 37117251 PMC10147366

[ref19] De Filippis F. Paparo L. Nocerino R. Della Gatta G. Carucci L. Russo R.. (2021). Specific gut microbiome signatures and the associated pro-inflamatory functions are linked to pediatric allergy and acquisition of immune tolerance. Nat. Commun. 12:5958. doi: 10.1038/s41467-021-26266-z, PMID: 34645820 PMC8514477

[ref20] Deng L. Wojciech L. Png C. W. Koh E. Y. Aung T. T. Kioh D. Y. Q.. (2022). Experimental colonization with Blastocystis ST4 is associated with protective immune responses and modulation of gut microbiome in a DSS-induced colitis mouse model. Cell. Mol. Life Sci. 79:245. doi: 10.1007/s00018-022-04271-9, PMID: 35435504 PMC9016058

[ref21] Diesner S. C. Bergmayr C. Pfitzner B. Assmann V. Krishnamurthy D. Starkl P.. (2016). A distinct microbiota composition is associated with protection from food allergy in an oral mouse immunization model. Clin. Immunol. 173, 10–18. doi: 10.1016/j.clim.2016.10.009, PMID: 27789346 PMC5464391

[ref22] Dominguez-Bello M. G. Godoy-Vitorino F. Knight R. Blaser M. J. (2019). Role of the microbiome in human development. Gut 68, 1108–1114. doi: 10.1136/gutjnl-2018-317503, PMID: 30670574 PMC6580755

[ref23] Dong P. Feng J. J. Yan D. Y. Lyu Y. J. Xu X. (2018). Early-life gut microbiome and cow's milk allergy- a prospective case - control 6-month follow-up study. Saudi J. Biol. Sci. 25, 875–880. doi: 10.1016/j.sjbs.2017.11.051, PMID: 30108435 PMC6088111

[ref24] Douglas G. M. Maffei V. J. Zaneveld J. R. Yurgel S. N. Brown J. R. Taylor C. M.. (2020). PICRUSt2 for prediction of metagenome functions. Nat. Biotechnol. 38, 685–688. doi: 10.1038/s41587-020-0548-6, PMID: 32483366 PMC7365738

[ref25] Duan C. Ma L. Yu J. Sun Y. Liu L. Ma F.. (2023). Oral administration of *Lactobacillus plantarum* JC7 alleviates OVA-induced murine food allergy through immunoregulation and restoring disordered intestinal microbiota. Eur. J. Nutr. 62, 685–698. doi: 10.1007/s00394-022-03016-5, PMID: 36194269 PMC9530419

[ref26] Dzidic M. Abrahamsson T. R. Artacho A. Collado M. C. Mira A. Jenmalm M. C. (2018). Oral microbiota maturation during the first 7 years of life in relation to allergy development. Allergy 73, 2000–2011. doi: 10.1111/all.13449, PMID: 29602225

[ref27] Edgar R. C. (2013). UPARSE: highly accurate OTU sequences from microbial amplicon reads. Nat. Methods 10, 996–998. doi: 10.1038/nmeth.2604, PMID: 23955772

[ref28] Escapa I. F. Chen T. Huang Y. Gajare P. Dewhirst F. E. Lemon K. P. (2018). New insights into human nostril microbiome from the expanded human oral microbiome database (eHOMD): a resource for the microbiome of the human aerodigestive tract. Msystems 3, 10–1128. doi: 10.1128/mSystems.00187-18, PMID: 30534599 PMC6280432

[ref29] Fazlollahi M. Chun Y. Grishin A. Wood R. A. Burks A. W. Dawson P.. (2018). Early-life gut microbiome and egg allergy. Allergy 73, 1515–1524. doi: 10.1111/all.13389, PMID: 29318631 PMC6436531

[ref30] Fernandes-Alnemri T. Wu J. Yu J. W. Datta P. Miller B. Jankowski W.. (2007). The pyroptosome: a supramolecular assembly of ASC dimers mediating inflammatory cell death via caspase-1 activation. Cell Death Differ. 14, 1590–1604. doi: 10.1038/sj.cdd.4402194, PMID: 17599095 PMC3345951

[ref31] Fu L. Peng J. Zhao S. Zhang Y. Su X. Wang Y. (2017). Lactic acid bacteria-specific induction of CD4(+)Foxp3(+)T cells ameliorates shrimp tropomyosin-induced allergic response in mice via suppression of mTOR signaling. Sci. Rep. 7:1987. doi: 10.1038/s41598-017-02260-8, PMID: 28512288 PMC5434066

[ref32] Fuks G. Elgart M. Amir A. Zeisel A. Turnbaugh P. J. Soen Y.. (2018). Combining 16S rRNA gene variable regions enables high-resolution microbial community profiling. Microbiome 6:17. doi: 10.1186/s40168-017-0396-x, PMID: 29373999 PMC5787238

[ref33] Fung T. C. Olson C. A. Hsiao E. Y. (2017). Interactions between the microbiota, immune and nervous systems in health and disease. Nat. Neurosci. 20, 145–155. doi: 10.1038/nn.4476, PMID: 28092661 PMC6960010

[ref34] García-Solache M. Rice L. B. (2019). The Enterococcus: a model of adaptability to its environment. Clin. Microbiol. Rev. 32, 10–128. doi: 10.1128/cmr.00058-18, PMID: 30700430 PMC6431128

[ref35] Georas S. N. Guo J. De Fanis U. Casolaro V. (2005). T-helper cell type-2 regulation in allergic disease. Eur. Respir. J. 26, 1119–1137. doi: 10.1183/09031936.05.00006005 16319345

[ref36] Goldberg M. R. Mor H. Magid Neriya D. Magzal F. Muller E. Appel M. Y.. (2020). Microbial signature in IgE-mediated food allergies. Genome Med. 12:92. doi: 10.1186/s13073-020-00789-4, PMID: 33109272 PMC7592384

[ref37] Han R. Yue J. Lin H. Du N. Wang J. Wang S.. (2021). Salivary microbiome variation in early childhood caries of children 3-6 years of age and its association with Iron deficiency Anemia and extrinsic black stain. Front. Cell. Infect. Microbiol. 11:628327. doi: 10.3389/fcimb.2021.628327, PMID: 33869076 PMC8044945

[ref38] Hanchi H. Mottawea W. Sebei K. Hammami R. (2018). The genus Enterococcus: between probiotic potential and safety concerns-an update. Front. Microbiol. 9:1791. doi: 10.3389/fmicb.2018.01791, PMID: 30123208 PMC6085487

[ref39] Ho H. E. Bunyavanich S. (2018). Role of the microbiome in food allergy. Curr Allergy Asthma Rep 18:27. doi: 10.1007/s11882-018-0780-z 29623445

[ref40] Ho H. E. Chun Y. Jeong S. Jumreornvong O. Sicherer S. H. Bunyavanich S. (2021). Multidimensional study of the oral microbiome, metabolite, and immunologic environment in peanut allergy. J. Allergy Clin. Immunol. 148, 627–632. doi: 10.1016/j.jaci.2021.03.028 33819506 PMC8355025

[ref41] Hufnagl K. Pali-Schöll I. Roth-Walter F. Jensen-Jarolim E. (2020). Dysbiosis of the gut and lung microbiome has a role in asthma. Semin. Immunopathol. 42, 75–93. doi: 10.1007/s00281-019-00775-y, PMID: 32072252 PMC7066092

[ref42] Jin B. Y. Li Z. Xia Y. N. Li L. X. Zhao Z. X. Li X. Y.. (2021). Probiotic interventions alleviate food allergy symptoms correlated with cesarean section: a murine model. Front. Immunol. 12:741371. doi: 10.3389/fimmu.2021.741371 34650564 PMC8505808

[ref43] Johnson J. S. Spakowicz D. J. Hong B. Y. Petersen L. M. Demkowicz P. Chen L.. (2019). Evaluation of 16S rRNA gene sequencing for species and strain-level microbiome analysis. Nat. Commun. 10:5029. doi: 10.1038/s41467-019-13036-1, PMID: 31695033 PMC6834636

[ref44] Johnson V. J. Yucesoy B. Luster M. I. (2005). Prevention of IL-1 signaling attenuates airway hyperresponsiveness and inflammation in a murine model of toluene diisocyanate-induced asthma. J. Allergy Clin. Immunol. 116, 851–858. doi: 10.1016/j.jaci.2005.07.008, PMID: 16210060

[ref45] Kil D. Y. Swanson K. S. (2011). Companion animals symposium: role of microbes in canine and feline health. J. Anim. Sci. 89, 1498–1505. doi: 10.2527/jas.2010-3498, PMID: 21036940

[ref46] Kim D. H. Jung W. S. Kim M. E. Lee H. W. Youn H. Y. Seon J. K.. (2014). Genistein inhibits pro-inflammatory cytokines in human mast cell activation through the inhibition of the ERK pathway. Int. J. Mol. Med. 34, 1669–1674. doi: 10.3892/ijmm.2014.1956, PMID: 25319548

[ref47] Kim W. G. Kang G. D. Kim H. I. Han M. J. Kim D. H. (2019). *Bifidobacterium longum* IM55 and *Lactobacillus plantarum* IM76 alleviate allergic rhinitis in mice by restoring Th2/Treg imbalance and gut microbiota disturbance. Benef. Microbes 10, 55–67. doi: 10.3920/bm2017.0146, PMID: 30465441

[ref48] Kim E. G. Leem J. S. Baek S. M. Kim H. R. Kim K. W. Kim M. N.. (2022). Interleukin-18 receptor α modulates the T cell response in food allergy. Allergy Asthma Immunol Res 14, 424–438. doi: 10.4168/aair.2022.14.4.424, PMID: 35837825 PMC9293601

[ref49] Lee K. H. Guo J. Song Y. Ariff A. O'Sullivan M. Hales B.. (2021). Dysfunctional gut microbiome networks in childhood IgE-mediated food allergy. Int. J. Mol. Sci. 22:2079. doi: 10.3390/ijms22042079, PMID: 33669849 PMC7923212

[ref50] Leibovitzh H. Lee S. H. Xue M. Raygoza Garay J. A. Hernandez-Rocha C. Madsen K. L.. (2022). Altered gut microbiome composition and function are associated with gut barrier dysfunction in healthy relatives of patients with Crohn's disease. Gastroenterology 163, 1364–1376. doi: 10.1053/j.gastro.2022.07.004, PMID: 35850197

[ref51] Lin N. Chi H. Guo Q. Liu Z. Ni L. (2024). Notch signaling inhibition alleviates allergies caused by Antarctic krill tropomyosin through improving Th1/Th2 imbalance and modulating gut microbiota. Food Secur. 13:1144. doi: 10.3390/foods13081144, PMID: 38672818 PMC11048830

[ref52] Liston A. Masters S. L. (2017). Homeostasis-altering molecular processes as mechanisms of inflammasome activation. Nat. Rev. Immunol. 17, 208–214. doi: 10.1038/nri.2016.151, PMID: 28163301

[ref53] Ma H. Shu Q. Li Z. Song X. Xu H. (2023). Formaldehyde aggravates allergic contact dermatitis by facilitating NLRP3 inflammasome activation in macrophages. Int. Immunopharmacol. 117:109904. doi: 10.1016/j.intimp.2023.109904, PMID: 36827924

[ref54] Malamud D. (1992). Saliva as a diagnostic fluid. BMJ 305, 207–208. doi: 10.1136/bmj.305.6847.207, PMID: 1290500 PMC1882670

[ref55] Marrs T. Jo J. H. Perkin M. R. Rivett D. W. Witney A. A. Bruce K. D.. (2021). Gut microbiota development during infancy: impact of introducing allergenic foods. J. Allergy Clin. Immunol. 147, 613–621. doi: 10.1016/j.jaci.2020.09.042, PMID: 33551026 PMC9169695

[ref56] Matsui S. Kataoka H. Tanaka J. I. Kikuchi M. Fukamachi H. Morisaki H.. (2019). Dysregulation of intestinal microbiota elicited by food allergy induces IgA-mediated oral dysbiosis. Infect. Immun. 88, 10–1128. doi: 10.1128/iai.00741-19, PMID: 31611274 PMC6921656

[ref57] Noval Rivas M. Burton O. T. Wise P. Zhang Y. Q. Hobson S. A. Garcia Lloret M.. (2013). A microbiota signature associated with experimental food allergy promotes allergic sensitization and anaphylaxis. J. Allergy Clin. Immunol. 131, 201–212. doi: 10.1016/j.jaci.2012.10.026, PMID: 23201093 PMC3860814

[ref58] Nowak-Wegrzyn A. Szajewska H. Lack G. (2017). Food allergy and the gut. Nat. Rev. Gastroenterol. Hepatol. 14, 241–257. doi: 10.1038/nrgastro.2016.187 27999436

[ref59] Nwaru B. I. Sheikh A. (2012). Risk factors for the development of egg allergy: progress to date and future directions. Allergy 67, 1325–1326. doi: 10.1111/all.12026, PMID: 23046159

[ref60] Pascal M. Perez-Gordo M. Caballero T. Escribese M. M. Lopez Longo M. N. Luengo O.. (2018). Microbiome and allergic diseases. Front Immunol 9:1584. doi: 10.3389/fimmu.2018.01584, PMID: 30065721 PMC6056614

[ref61] Pérez-Losada M. Castro-Nallar E. Laerte Boechat J. Delgado L. Azenha Rama T. Berrios-Farías V.. (2023). The oral bacteriomes of patients with allergic rhinitis and asthma differ from that of healthy controls. Front. Microbiol. 14:1197135. doi: 10.3389/fmicb.2023.1197135, PMID: 37440882 PMC10335798

[ref62] Pootakham W. Mhuantong W. Yoocha T. Putchim L. Sonthirod C. Naktang C.. (2017). High resolution profiling of coral-associated bacterial communities using full-length 16S rRNA sequence data from PacBio SMRT sequencing system. Sci. Rep. 7:2774. doi: 10.1038/s41598-017-03139-4, PMID: 28584301 PMC5459821

[ref63] Prince B. T. Mandel M. J. Nadeau K. Singh A. M. (2015). Gut microbiome and the development of food allergy and allergic disease. Pediatr. Clin. N. Am. 62, 1479–1492. doi: 10.1016/j.pcl.2015.07.007, PMID: 26456445 PMC4721650

[ref64] Savage J. H. Matsui E. C. Skripak J. M. Wood R. A. (2007). The natural history of egg allergy. J. Allergy Clin. Immunol. 120, 1413–1417. doi: 10.1016/j.jaci.2007.09.040 18073126

[ref65] Schei K. Simpson M. R. Øien T. Salamati S. Rudi K. Ødegård R. A. (2021). Allergy-related diseases and early gut fungal and bacterial microbiota abundances in children. Clin. Transl. Allergy 11:e12041. doi: 10.1002/clt2.12041, PMID: 34194728 PMC8238386

[ref66] Schloss P. D. Westcott S. L. Ryabin T. Hall J. R. Hartmann M. Hollister E. B.. (2009). Introducing mothur: open-source, platform-independent, community-supported software for describing and comparing microbial communities. Appl. Environ. Microbiol. 75, 7537–7541. doi: 10.1128/aem.01541-09, PMID: 19801464 PMC2786419

[ref67] Segata N. Izard J. Waldron L. Gevers D. Miropolsky L. Garrett W. S.. (2011). Metagenomic biomarker discovery and explanation. Genome Biol. 12:R60. doi: 10.1186/gb-2011-12-6-r60, PMID: 21702898 PMC3218848

[ref68] Simpson J. L. Phipps S. Baines K. J. Oreo K. M. Gunawardhana L. Gibson P. G. (2014). Elevated expression of the NLRP3 inflammasome in neutrophilic asthma. Eur. Respir. J. 43, 1067–1076. doi: 10.1183/09031936.00105013, PMID: 24136334

[ref69] Stefka A. T. Feehley T. Tripathi P. Qiu J. McCoy K. Mazmanian S. K.. (2014). Commensal bacteria protect against food allergen sensitization. Proc. Natl. Acad. Sci. USA 111, 13145–13150. doi: 10.1073/pnas.1412008111, PMID: 25157157 PMC4246970

[ref70] Tian X. Fan R. He H. Cui Q. Liang X. Liu Q.. (2022). *Bifidobacterium animalis* KV9 and *Lactobacillus vaginalis* FN3 alleviated β-lactoglobulin-induced allergy by modulating dendritic cells in mice. Front. Immunol. 13:992605. doi: 10.3389/fimmu.2022.992605, PMID: 36238281 PMC9552907

[ref71] Vanderhoof J. A. Young R. J. (2003). Role of probiotics in the management of patients with food allergy. Ann. Allergy Asthma Immunol. 90, 99–103. doi: 10.1016/s1081-1206(10)61669-9 12839122

[ref72] Wang Q. Garrity G. M. Tiedje J. M. Cole J. R. (2007). Naive Bayesian classifier for rapid assignment of rRNA sequences into the new bacterial taxonomy. Appl. Environ. Microbiol. 73, 5261–5267. doi: 10.1128/aem.00062-07, PMID: 17586664 PMC1950982

[ref73] Wang L. Li W. Xin S. Wu S. Peng C. Ding H.. (2023). Soybean glycinin and β-conglycinin damage the intestinal barrier by triggering oxidative stress and inflammatory response in weaned piglets. Eur. J. Nutr. 62, 2841–2854. doi: 10.1007/s00394-023-03188-8, PMID: 37358571

[ref74] Wang Y. Ouyang M. Gao X. Wang S. Fu C. Zeng J.. (2020). Phocea, Pseudoflavonifractor and *Lactobacillus intestinalis*: three potential biomarkers of gut microbiota that affect progression and complications of obesity-induced type 2 diabetes mellitus. Diabetes Metab Syndr Obes 3, 835–850. doi: 10.2147/dmso.S240728, PMID: 32256098 PMC7090210

[ref75] Wang Y. Zhang D. Liu T. Wang J. F. Wu J. X. Zhao J. P.. (2021). FSTL1 aggravates OVA-induced inflammatory responses by activating the NLRP3/IL-1β signaling pathway in mice and macrophages. Inflamm. Res. 70, 777–787. doi: 10.1007/s00011-021-01475-w, PMID: 34076707

[ref76] Wang Z. Zhang J. Yuan J. Min F. Gao J. Liu W.. (2024). Oral administration of egg ovalbumin allergen induces dysregulation of tryptophan metabolism in sensitized BALB/c mice. Food Funct. 15, 4375–4388. doi: 10.1039/d3fo05300h, PMID: 38546528

[ref77] Weisburg W. G. Barns S. M. Pelletier D. A. Lane D. J. (1991). 16S ribosomal DNA amplification for phylogenetic study. J. Bacteriol. 173, 697–703. doi: 10.1128/jb.173.2.697-703.1991, PMID: 1987160 PMC207061

[ref78] Wilkins L. J. Monga M. Miller A. W. (2019). Defining dysbiosis for a cluster of chronic diseases. Sci. Rep. 9:12918. doi: 10.1038/s41598-019-49452-y, PMID: 31501492 PMC6733864

[ref79] Wood R. A. (2016). Advances in food allergy in 2015. J. Allergy Clin. Immunol. 138, 1541–1547. doi: 10.1016/j.jaci.2016.10.002, PMID: 27931535

[ref80] Xiao J. Fiscella K. A. Gill S. R. (2020). Oral microbiome: possible harbinger for children's health. Int. J. Oral Sci. 12:12. doi: 10.1038/s41368-020-0082-x, PMID: 32350240 PMC7190716

[ref81] Xu J. Ye Y. Ji J. Sun J. Wang J. S. Sun X. (2022). Untargeted Metabolomic profiling reveals changes in gut microbiota and mechanisms of its regulation of allergy in OVA-sensitive BALB/c mice. J. Agric. Food Chem. 70, 3344–3356. doi: 10.1021/acs.jafc.1c07482, PMID: 35232013

[ref82] Zhang L. Chun Y. Ho H. E. Arditi Z. Lo T. Sajja S.. (2022). Multiscale study of the oral and gut environments in children with high- and low-threshold peanut allergy. J. Allergy Clin. Immunol. 150, 714–720. doi: 10.1016/j.jaci.2022.04.026, PMID: 35550149 PMC9463091

