# Supplementary material for: Impact of ovalbumin allergy on oral and gut microbiome dynamics in 6-week-old BALB/c mice
Source: Front Microbiol. 2024 Sep 3;15:1439452. doi: 10.3389/fmicb.2024.1439452 (PMC11406088; doi:10.3389/fmicb.2024.1439452)
Supplement: Supplementary file 1 [file Data_Sheet_1.docx]

Supplementary Material

# Supplementary Figures and Tables

## Supplementary Figure


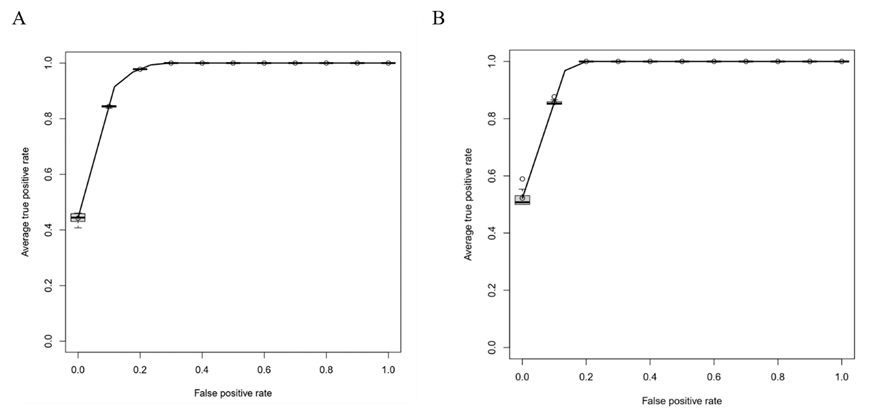


**Supplementary Figure S1.** The ROC curve of the classifier that trades off the rate of true positives against the rate of false positives. (A)Fecal samples; (B)Saliva samples.

## Supplementary Tables

**TableS 1**. Differences in salivary flora between species at the phylum level between the two groups.

| **Phylum** | **Con** | | **Ova** | | ***P*_value** |
| --- | --- | --- | --- | --- | --- |
|  | Mean (%) | Sd (%) | Mean (%) | Sd (%) |  |
| *Proteobacteria* | 44.03 | 21.52 | 54.59 | 21.26 | 0.4433 |
| *Firmicutes* | 53.95 | 21.22 | 38.92 | 17.31 | 0.2502 |
| *Bacteroidota* | 0.5677 | 0.4459 | 1.607 | 2.131 | 0.1599 |
| *Planctomycetota* | 0.2279 | 0.2586 | 1.47 | 1.69 | 0.05502 |
| *Actinobacteria* | 0.1427 | 0.139 | 1.085 | 1.257 | 0.09596 |
| *Acidobacteria* | 0.09053 | 0.159 | 0.9101 | 0.8029 | **0.03654** |
| *Cyanobacteria* | 0.6439 | 1.092 | 0.3046 | 0.7376 | 0.7903 |
| *Chloroflexi* | 0.04367 | 0.05746 | 0.6747 | 0.585 | **0.01166** |
| *unclassified_d__Bacteria* | 0.1518 | 0.2268 | 0.1454 | 0.07371 | 0.37 |
| *Nitrospirae* | 0.01118 | 0.01582 | 0.115 | 0.1293 | **0.04971** |
| *Deinococcus-Thermus* | 0.06071 | 0.112 | 0.06337 | 0.0803 | 0.6823 |
| *Verrucomicrobia* | 0.03675 | 0.08626 | 0.03249 | 0.01621 | 0.06732 |
| *Armatimonadetes* | 0.0005326 | 0.001409 | 0.03728 | 0.04581 | 0.07395 |
| *Deferribacteres* | 0.02769 | 0.07327 | 0 | 0 | 0.3914 |
| *Ignavibacteriae* | 0.0005326 | 0.001409 | 0.0245 | 0.02183 | **0.007644** |
| *Synergistetes* | 0.001598 | 0.004227 | 0.009053 | 0.0172 | 0.477 |
| *Gemmatimonadetes* | 0.007988 | 0.01467 | 0 | 0 | 0.173 |
| *Candidatus_Melainabacteria* | 0.004793 | 0.01268 | 0 | 0 | 0.3914 |
| *Spirochaetes* | 0.0005326 | 0.001409 | 0.00426 | 0.008454 | 0.477 |
| *Fusobacteria* | 0.002663 | 0.007045 | 0 | 0 | 0.3914 |
| *Elusimicrobia* | 0 | 0 | 0.002663 | 0.007045 | 0.3914 |
| *Lentisphaerae* | 0 | 0 | 0.00213 | 0.005636 | 0.3914 |

values in bold are statistically significant.

**TableS 2**. Differentially abundant gut microbiome species between mice with OVA-allergy and healthy controls.

| **Species name** | **Strength groups (Ova/Con)** | ***P*_value** |
| --- | --- | --- |
| *s__Achromobacter_spanius_g__norank* | Ova | **0.0072** |
| *s__uncultured_bacterium_g__Harryflintia* | Ova | **0.0200** |
| *s__Acinetobacter_sp__CIP_56_2* | Ova | **0.0072** |
| *s__unclassified_g__norank_f__Ruminococcaceae* | Ova | **0.0374** |
| *s__Candidatus_Arthromitus_sp__SFB-mouse-Japan* | Ova | **0.0092** |
| *s__Agrobacterium_radiobacter* | Ova | **0.0463** |
| *s__uncultured_Clostridiales_bacterium_g__norank_f__Lachnospiraceae* | Ova | **0.0072** |
| *s__unclassified_f__Lachnospiraceae* | Ova | **0.0181** |
| *s__unclassified_g__norank_f__Erysipelotrichaceae* | Ova | **0.0324** |
| *s__unclassified_g__Gemella* | Ova | **0.0338** |
| *s__unclassified_g__Enterococcus* | Con | **0.0021** |
| *s__Lactobacillus_intestinalis* | Con | **0.0343** |
| *s__uncultured_Bacteroidales_bacterium_g__norank_f__Muribaculaceae* | Con | **0.0433** |
| *s__unclassified_g__Lactobacillus* | Con | **0.0103** |
| *s__Metamycoplasma_sualvi* | Con | **0.0269** |
| *s__uncultured_rumen_bacterium_g__norank_f__norank_o__Clostridia_UCG-014* | Con | **0.0386** |
| *s__unclassified_g__norank_f__Muribaculaceae* | Con | **0.0433** |
| *s__unidentified_rumen_bacterium_JW32* | Con | **0.0386** |

values in bold are statistically significant.

**TableS 3**. Differentially abundant oral microbiome species between mice with OVA-allergy and healthy controls.

| **Species name** | **Strength groups (Ova/Con)** | ***P*_value** |
| --- | --- | --- |
| *s__unclassified_g__Staphylococcus* | Con | **0.0127** |
| *s__Streptococcus_hyointestinalis* | Con | **0.0344** |
| *s__unclassified_g__Dechloromonas* | Con | **0.0222** |
| *s__unclassified_g__Tissierella* | Ova | **0.0311** |
| *s__unclassified_o__Bacteroidales* | Ova | **0.0176** |
| *s__unclassified_f__Tissierellaceae* | Ova | **0.0176** |
| *s__Proteiniclasticum_ruminis* | Ova | **0.0167** |
| *s__unclassified_o__Eubacteriales* | Ova | **0.0168** |
| *s__unclassified_f__Gemmataceae* | Ova | **0.0238** |
| *s__Guggenheimella_bovis* | Ova | **0.0311** |
| *s__Romboutsia_timonensis* | Ova | **0.0474** |
| *s__unclassified_f__Sterolibacteriaceae* | Ova | **0.0330** |
| *s__Proteiniborus_ethanoligenes* | Ova | **0.0021** |
| *s__unclassified_f__Caldilineaceae* | Ova | **0.0024** |
| *s__unclassified_f__Oscillospiraceae* | Ova | **0.0252** |
| *s__Stenotrophobacter_terrae* | Ova | **0.0333** |
| *s__unclassified_f__Clostridiaceae* | Ova | **0.0333** |
| *s__unclassified_g__Brevitalea* | Ova | **0.0283** |
| *s__Actinomarinicola_tropica* | Ova | **0.0344** |
| *s__unclassified_f__Iamiaceae* | Ova | **0.0156** |
| *s__unclassified_g__Stenotrophobacter* | Ova | **0.0344** |
| *s__unclassified_c__Betaproteobacteria* | Ova | **0.0330** |
| *s__unclassified_g__Bradyrhizobium* | Ova | **0.0344** |
| *s__unclassified_g__Levilactobacillus* | Ova | **0.0344** |
| *s__Tissierella_carlieri* | Ova | **0.0467** |
| *s__unclassified_p__Chloroflexi* | Ova | **0.0250** |
| *s__Sinanaerobacter_chloroacetimidivorans* | Ova | **0.0108** |
| *s__Hyphomicrobium_facile* | Ova | **0.0115** |
| *s__Stenotrophobacter_roseus* | Ova | **0.0461** |
| *s__unclassified_c__Planctomycetia* | Ova | **0.0344** |
| *s__unclassified_f__Anaerolineaceae* | Ova | **0.0311** |
| *s__unclassified_f__Peptoniphilaceae* | Ova | **0.0156** |
| *s__unclassified_c__Deltaproteobacteria* | Ova | **0.0283** |
| *s__unclassified_f__Planctomycetaceae* | Ova | **0.0028** |
| *s__unclassified_o__Pirellulales* | Ova | **0.0222** |
| *s__unclassified_f__Rikenellaceae* | Ova | **0.0091** |
| *s__Defluviimonas_aquaemixtae* | Ova | **0.0283** |
| *s__unclassified_c__Actinomycetia* | Ova | **0.0030** |
| *s__Nordella_oligomobilis* | Ova | **0.0250** |
| *s__Nitrospira_moscoviensis* | Ova | **0.0452** |
| *s__Lentimicrobium_saccharophilum* | Ova | **0.0344** |
| *s__unclassified_c__Alphaproteobacteria* | Ova | **0.0250** |
| *s__unclassified_f__Azonexaceae* | Ova | **0.0156** |
| *s__Lignipirellula_cremea* | Ova | **0.0250** |
| *s__Ilumatobacter_fluminis* | Ova | **0.0283** |
| *s__unclassified_g__Terrimonas* | Ova | **0.0461** |
| *s__Trichococcus_flocculiformis* | Ova | **0.0115** |
| *s__unclassified_g__Ilumatobacter* | Ova | **0.0402** |
| *s__Nitrolancea_hollandica* | Ova | **0.0033** |
| *s__Aquihabitans_daechungensis* | Ova | **0.0196** |
| *s__unclassified_c__Anaerolineae* | Ova | **0.0344** |
| *s__unclassified_g__Acetobacterium* | Ova | **0.0250** |
| *s__unclassified_f__Haliscomenobacteraceae* | Ova | **0.0283** |
| *s__unclassified_o__Ignavibacteriales* | Ova | **0.0196** |
| *s__Mesorhizobium_huakuii* | Ova | **0.0052** |
| *s__unclassified_f__Alcaligenaceae* | Ova | **0.0250** |
| *s__unclassified_f__Erysipelotrichaceae* | Ova | **0.0092** |
| *s__Stenotrophobacter_namibiensis* | Ova | **0.0244** |
| *s__unclassified_g__Gemmata* | Ova | **0.0250** |
| *s__unclassified_f__Prolixibacteraceae* | Ova | **0.0250** |
| *s__Pseudorhodoplanes_sinuspersici* | Ova | **0.0250** |
| *s__unclassified_g__Devosia* | Ova | **0.0283** |
| *s__Zeimonas_arvi* | Ova | **0.0250** |
| *s__Planctopirus_ephydatiae* | Ova | **0.0250** |
| *s__Quisquiliibacterium_transsilvanicum* | Ova | **0.0250** |
| *s__Andreesenia_angusta* | Ova | **0.0250** |

values in bold are statistically significant.

**TableS 4**. KEGG pathway prediction in fecal samples of OVA-sensitive mice.

| **Pathways of level 3 in fecal** | **Fold Change (Ova/Con)** | ***P*_value** |
| --- | --- | --- |
| Fatty acid metabolism | 1.13148 | **0.02707** |
| Bacterial chemotaxis | 3.34444 | **0.01115** |
| Flagellar assembly | 3.74482 | **0.01736** |
| Salmonella infection | 1.1395 | **0.00443** |
| NOD-like receptor signaling pathway | 1.24452 | **0.002171** |
| Legionellosis | 1.20352 | **0.01434** |
| Insect hormone biosynthesis | 2.92688 | **0.03401** |
| Parkinson disease | 2.71844 | **0.02104** |
| Non-alcoholic fatty liver disease (NAFLD) | 2.70535 | **0.01716** |
| Cardiac muscle contraction | 3.82222 | **0.02093** |
| Sphingolipid signaling pathway | 87.56668 | **0.03391** |
| Metabolic pathways | 0.98674 | **0.04196** |
| Galactose metabolism | 0.89368 | **0.0318** |
| Ubiquinone and other terpenoid-quinone biosynthesis | 0.75351 | **0.04452** |
| Primary immunodeficiency | 0.82797 | **0.009973** |
| Type I diabetes mellitus | 0.83296 | **0.0005411** |
| Pathogenic Escherichia coli infection | 0.92972 | **0.01563** |
| Primary bile acid biosynthesis | 0.7278 | **0.03712** |
| Nonribosomal peptide structures | 0.62782 | **0.03745** |

values in bold are statistically significant.

**TableS 5**. KEGG pathway prediction in saliva samples of OVA-sensitive mice.

| **Pathways of level 3 in saliva** | **Fold Change (Ova/Con)** | ***P*_value** |
| --- | --- | --- |
| Oxidative phosphorylation | 1.06374 | **0.04269** |
| Citrate cycle (TCA cycle) | 1.06165 | **0.02107** |
| Histidine metabolism | 1.06972 | **0.01173** |
| Glucosinolate biosynthesis | 1.08775 | **0.01391** |
| NOD-like receptor signaling pathway | 1.11304 | **0.01358** |
| Nitrotoluene degradation | 1.25988 | **0.0392** |
| Furfural degradation | 10.87987 | **0.01846** |
| Indole alkaloid biosynthesis | 6.0311 | **0.04298** |
| Glycosphingolipid biosynthesis - lacto and neolacto series | 100.66057 | **0.0398** |
| Olfactory transduction | 9.58173 | **0.03752** |
| Phototransduction - fly | 14.29279 | **0.0369** |
| Neurotrophin signaling pathway | 14.29279 | **0.0369** |
| Circadian entrainment | 14.29279 | **0.0369** |
| ErbB signaling pathway | 14.29279 | **0.0369** |
| Glioma | 14.29279 | **0.0369** |

values in bold are statistically significant.
